# Polysaccharide Hydroxyapatite (Nano)composites and Their Biomedical Applications: An Overview of Recent Years

**DOI:** 10.1021/acsomega.4c02170

**Published:** 2024-07-02

**Authors:** Wanderson Barros Costa, Ana F. Félix Farias, Edson Cavalcanti Silva-Filho, Josy A. Osajima, Santiago Medina-Carrasco, Maria Del Mar Orta, Maria G. Fonseca

**Affiliations:** a Fuel and Materials Laboratory − NPE-LACOM, UFPB, 58051-085, João Pessoa, Paraiba, Brazil; b Interdisciplinary Laboratory for Advanced Materials − LIMAV, UFPI, 64049-550, Teresina, Piaui, Brazil; c SGI Laboratorio de Rayos X - Centro de Investigación, Tecnología e Innovación de la Universidad de Sevilla (CITIUS), 41012, Sevilla, Spain; d Departamento de Química Analítica, Facultad de Farmacia, Universidad de Sevilla, C/Profesor García, González 2, 41012 Sevilla, Spain

## Abstract

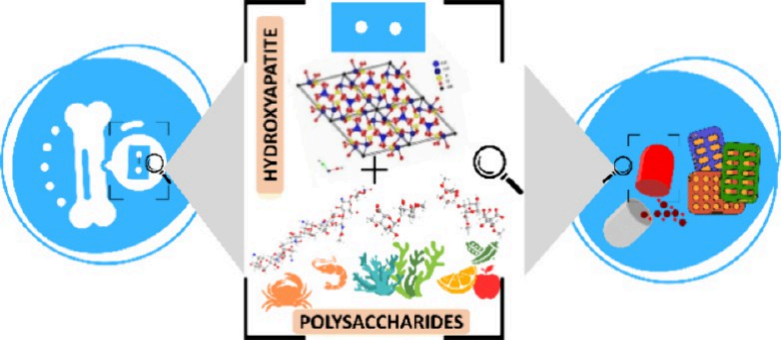

Hydroxyapatite can combine with polysaccharide originating biomaterials with special applications in the biomedical field. In this review, the synthesis of (nano)composites is discussed, focusing on natural polysaccharides such as alginate, chitosan, and pectin. In this way, advances in recent years in the development of preparing materials are revised and discussed. Therefore, an overview of the recent synthesis and applications of polyssacharides@hydroxyapatites is presented. Several studies based on chitosan@hydroxyapatite combined with other inorganic matrices are highlighted, while pectin@hydroxyapatite is present in a smaller number of reports. Biomedical applications as drug carriers, adsorbents, and bone implants are discussed, combining their dependence with the nature of interactions on the molecular scale and the type of polysaccharides used, which is a relevant aspect to be explored.

## 1 Introduction

Biomaterials research has been a field of great interest in the last two decades, playing an important role in relation to healthcare outcomes, increasing longevity, and improving health and quality of life through medical applications and biological medical devices for human use based on genes, cells, and/or tissue engineering, and also advanced therapies.^1^

Incorporation of organic matrices into inorganic materials allows the formation of organic@inorganic hybrid materials and favors the control of spatial organization of these materials, often found in the living body, especially in hard tissue, allowing the development of new biomaterials with tailored structure and properties.^2−4^ Structures such as scaffolds are examples of hybrid systems that have attracted attention in recent decades, since they involve two distinct phases and due to the specific advantages conferred by the attributed polymer and ceramic properties, making it possible to obtain hybrid material with better physical properties, for example, flexibility.^3−5^ In bone tissue engineering, the main purpose is to design a biocompatible porous scaffold with high interconnectivity that can provide an appropriate environment for delivery.^3,5^ Another advantage of scaffolds is that they have great potential to obtain systems that carry molecules with therapeutic properties.^−8^ The use of scaffolds is an essential factor for hard tissue regeneration by providing the desired surface and space for cells to attach, proliferate, migrate, and differentiate to organize normal bone tissue.^6^

Generally, organic matrices can be, e.g., polysaccharides that are used during the synthesis of organic/inorganic hybrid materials.^4^ Polysaccharides such as chitosan have been widely studied for bone tissue engineering applications due to their multiple advantages such as easy functionalization for drug delivery purposes, hydrophilic behavior, nonharmful degradation by-products, ability to accelerate wound healing and support adhesion, proliferation, and differentiation of mesenchymal stem cells into bone cells.^9^

The mineralization of these organic@inorganic hybrids can be favored by polymeric matrices and therefore imitate the structure present in living organisms.^3,9^ Another aspect is that inorganic materials present good synergism with organic matrices, as they can also improve the mechanical and chemical properties of the resulting materials.^10,11^

A classic example of hybrid materials easily found in living organisms, especially in hard tissues, are bones and teeth, where they are mainly made of hydroxyapatite and collagen fibers.^12−14^ These biomaterials can act, for example, as new artificial bone substitutes, showing bioactivity and mechanical performance analogous to natural bone.^2,15^

Studies have shown that the addition of inorganic fillers, such as calcium phosphates, mainly hydroxyapatite, to polysaccharide matrices can increase the biocompatibility, osteoconductivity, and bioactivity of the material obtained.^11^ Some reports have observed that the incorporation of hydroxyapatite with polysaccharides allows control of the morphological, structural, and mechanical properties of composites, improving their applications.^16,17^

Since the mechanical properties and interactions between the hydroxyapatite crystal and the polymer chain are affected by the molecular mass of the polysaccharide, therefore, the polysaccharide matrix influences the control of nucleation and growth of hydroxyapatite crystals when used in the formation of hybrid systems.^17,18^ This occurs because polymer chains modulate the morphology of hydroxyapatite *in vitro*, as well as its occurrence *in vivo*.^2^

Therefore, different types of natural polysaccharides have been used to obtain hybrid materials using hydroxyapatite as a ceramic reinforcement. Among polysaccharides, the following can be highlighted: alginate,^8,11,19,20^ chitosan,^9,21,22^ cellulose,^12^ and pectine.^23,24^

In this way, the synthesis of hydroxyapatite@polysaccharide bionanocomposites has been a promising target for study. In this sense, this review is based on the preparation of these materials for use in the biomedical field as vehicles for the adsorption or delivery systems of drugs and other bioactive molecules, bone implants, and other applications, e.g., antimicrobial agents. Special emphasis will be placed on the synthesis of these materials in addition to the understanding of the interactions on a molecular scale in the formation of these hybrid systems.

### 1.1 Research Planning

This review was carried out through a quantitative systematic review of specialized literature in the Web of Science, Pubmed, and Scopus databases, between 2012 and 2024. Exclusion criteria were reports published before 2012 and reviews and reports that do not deal with hydroxyapatite and polysaccharides.

The report selection process followed the rules of the Preferred Reporting Items for Systematic Reviews and Meta-Analyses (PRISMA). The objective of the technique is to improve the quality of systematic review protocols. Therefore, the study was divided into three stages: Initially, reports were identified in databases, in the second stage, screening was carried out according to exclusion criteria, and in the third moment, the eligibility of the investigated reports was carried out after reading them in the second stage.^25,26^

Table 1 summarizes the keywords and the total number of reports obtained. Searching on the Web of Science and Scopus platforms included journals and reviews, book chapters, and letters, while on the PubMed platform, articles were not selected by types.

**Table 1:** Terms Used in the Search and the Number of Publications (Article, Review Article, Book Chapters, and Letters) on Each Platform^a^

keywords	Web of Science	PubMed	Scopus
“Biomedical application” or biomedicine	54,951	117,027	1,279,973
Biomaterials or “biomaterials generation”	85,529	160,646	833,669
Polysaccharide	86,950	242,833	468,803
Hydroxyapatite or HAp	43,205	66,377	220,063
Hydroxyapatite and chitosan	2,706	14,975	69,873
Hydroxyapatite and alginate	894	9,334	34,966
Hydroxyapatite and pectin	62	1,580	5,993
Hydroxyapatite and adsorption or “Drug adsorption”	4,596	13,797	58,776
Hydroxyapatite and “bone tissue engineering”	3,684	13,011	48,350
Hydroxyapatite and “Drug delivery”	2,755	13,498	56,498
Hydroxyapatite and “Implant bone”	153	1,348	3,654
Hydroxyapatite and chitosan and adsorption or “drug adsorption”	779	7,739	28,656
Hydroxyapatite and chitosan and “bone tissue engineering”	665	7,575	28,260
Hydroxyapatite and chitosan and “drug delivery”	366	8,678	35,345
Hydroxyapatite and chitosan and “Implant bone”	4	309	901
Hydroxyapatite and alginate and adsorption or “Drug adsorption”	570	5,054	14,290
Hydroxyapatite and alginate and “bone tissue engineering”	187	5,251	16,651
Hydroxyapatite and alginate and “drug delivery”	141	5,743	20,543
Hydroxyapatite and alginate and “implant bone”	4	140	410
Hydroxyapatite and pectin and adsorption or “drug adsorption”	462	1,978	2,909
Hydroxyapatite and pectin and “bone tissue engineering”	9	717	2,364
Hydroxyapatite and pectin and “drug delivery”	8	1,099	3,928
Hydroxyapatite and pectin and “Implant bone”	0	20	42

a Date: January 2012 – April 2024.

A significant number of reports were published on each platform when generic search terms were applied. For example, using HYDROXYAPATITE or HAp, which include calcium phosphates and nanohydroxyapatite, the Web of Science platform presented over 43000 reports, since several research fields are in different areas for the same term.

**Figure 1:**
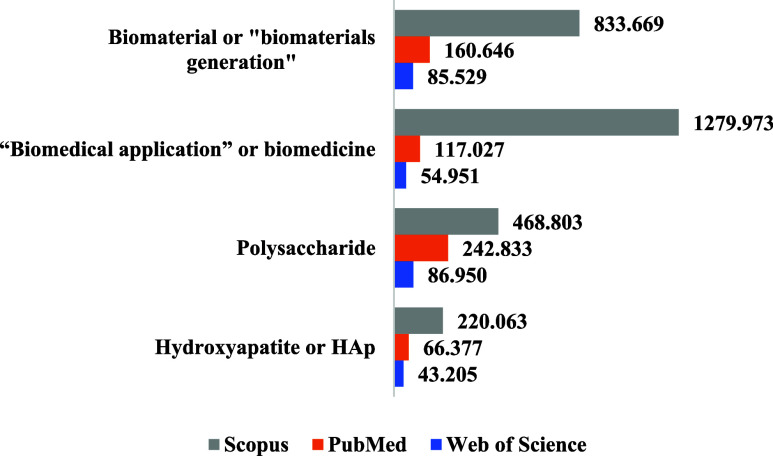
Number of publications with generic terms found on the Scopus, PubMed, and Web of Science platforms. Date: January 2012 – April 2024.

In this sense, the number of reports found with generic terms on the three platforms is presented in Figure 1. Data indicated that with all the keywords evaluated, a greater number of reports were in the Scopus platform, followed by PubMed and Web of Science. Like this, reports involving the keywords: Biomaterials or Biomaterials Generation can be included in publications identified with the use of the term “Biomedical application” or biomedicine.

**Figure 2:**
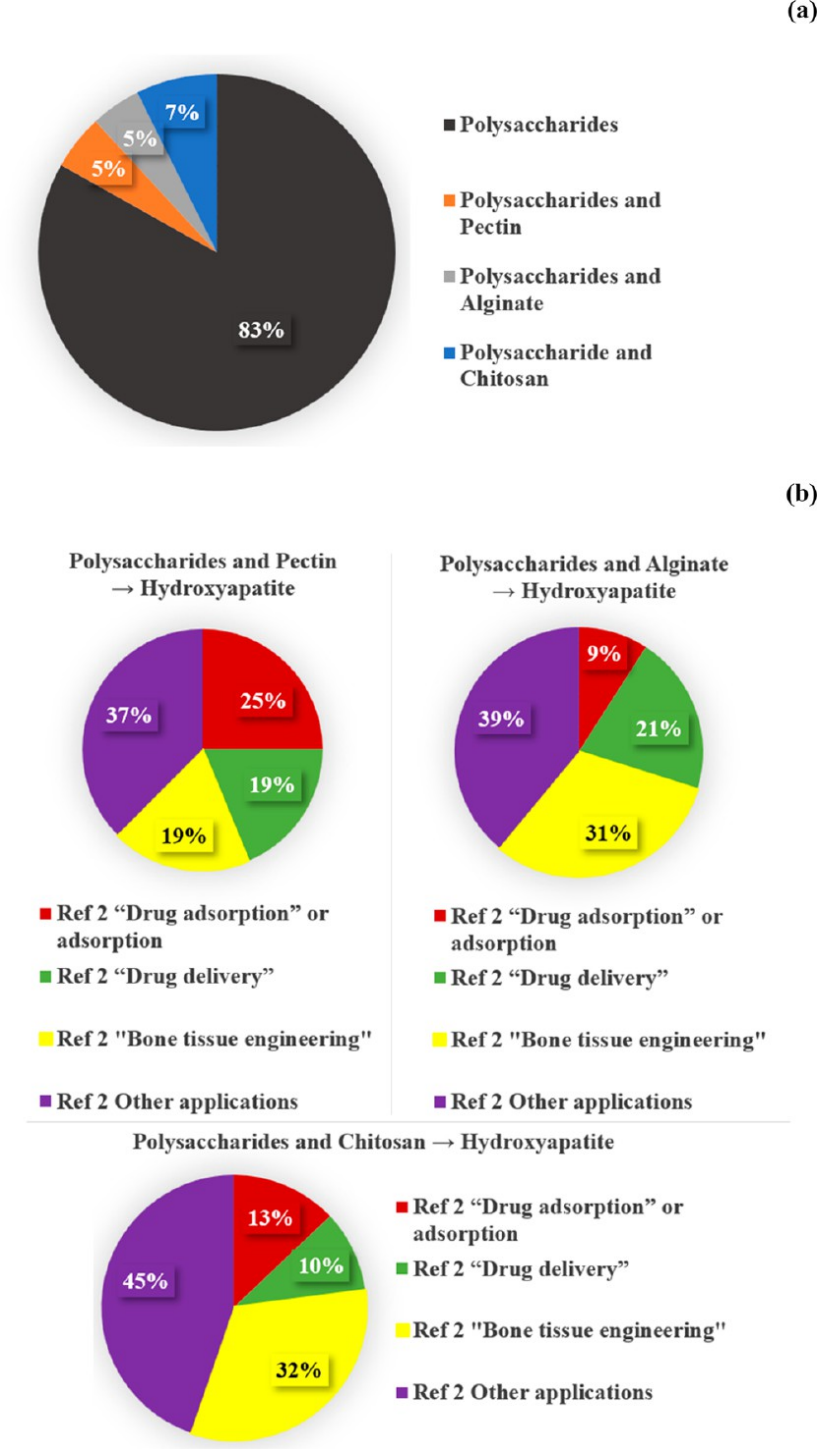
Number of publications found on the Web of Science Platform using the keywords polysaccharides (a) and their principal types as keywords, (b) refining to hydroxyapatite in Refinement 1 (Ref 1) followed by their applications in Refinement 2 (Ref 2). Date: January 2012 – April 2024.

The studies were found in the three search platforms by searching the term HYDROXYAPATITE or HAp; also, they can be included in the number of reports by using the keyword POLYSACCHARIDE, since a similar profile was observed. In this sense, during the evaluation of the reports presented in Table 1, more studies used the term “BONE TISSUE ENGINEERING” instead of “BONE IMPLANT” as a keyword in the application. Therefore, in a second moment of data processing, only studies found on the Web of Science platform were considered, initially using the most specific terms for POLYSACCHARIDES covered in this review (Figure 2a), and refining the term HYDROXYAPATITE or HAp, followed by another refinement with terms associated with applications: “DRUG ADSORPTION” or “ADSORPTION”, “DRUG DELIVERY”, and “BONE TISSUE ENGINEERING”, as given in Figure 2b.

Between reports on polysaccharides (Figure 2a), a higher number of reports on chitosan (7%) was obtained, following by alginate (5%) and pectin (5%). Therefore, 83% of the reports were carried out with other types of polysaccharides, for example, carrageenan, starch, hyaluronic acid, and others. For systems based on polysaccharides and hydroxyapatite (Figure 2b), when evaluated with respect to applications, 60% of the reports were carried out using systems based on chitosan, pectin, and alginate. In this sense, the biomaterials associated with the latter systems were chosen as criteria to be emphasized in this review.

Among the systems based on polysaccharides and hydroxyapatite (Figure 2b), evaluation in relation to applications in “Bone Tissue Engineering”, the systems based on chitosan presented 32%, alginate (31%), and pectin (19%).

For applications in drug adsorption or adsorption, the systems based on pectin and hydroxyapatite stand out as the most studied among the systems evaluated (25%), followed by systems with chitosan (13%) and alginate (9%). Finally, among reports, those obtained with alginate and hydroxyapatite involved a drug delivery process (21%), followed by pectin (19%) and chitosan (10%).

Based on these criteria, the present review emphasizes biomaterials obtained from hydroxyapatite (HAp) and polysaccharides, especially those systems based on chitosan, pectin, and alginate.

## 2 Biomaterials

Considering that the materials described in this review have biomedical applications, especially in tissue engineering, it is necessary to give a definition of biomaterials and a brief contextualization of the tissue engineering field.

Biomaterial and its possible synonym biomedical material were discussed in the Chengdu Conference on Definitions in Biomaterials at 2019, to aim to establish connections, differences, or its use as synonym.^27^ Therefore, the use of the term “Biomaterial” was discussed considering other seven related terms (biomaterial subgroups, biocompatibility and immune responses, degradation phenomena, regeneration, devices based on biomaterials, biomaterial delivery systems, and biotechnology based on biomaterials), and finally a simplified definition was proposed for biomaterial as a substance designed to assume a form that can direct, by controlling interactions with living systems, the course of any therapeutic or diagnostic procedure.^27^

**Figure 3:**
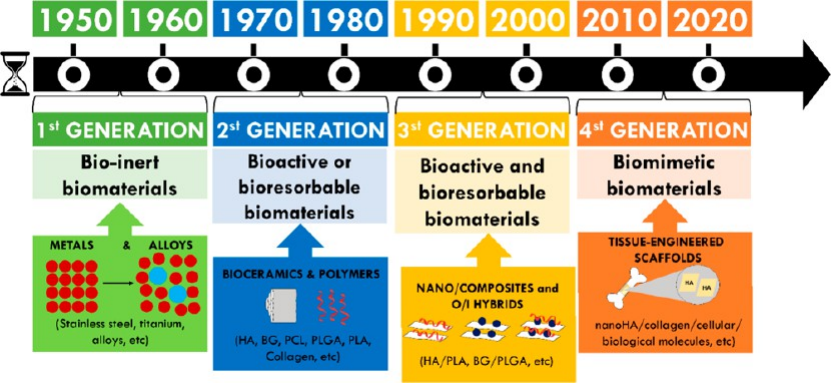
Evolution of biomaterials.

It means that “biomaterials” are body-compatible and used alone or in combination, modified, or designed as a device. In fact, a rapid evolution of biomaterials and their performance have been observed in recent years, since they can be used both in diagnosis and in treatment, being able to interact with biological systems and contribute to the correction of abnormalities, in the replacement of diseased or damaged parts in the body, or even in the healing of tissues.^28,29^ Biomaterials can have natural or synthetic origin and are classified according to their constituents, which can be metal or nonmetal. Therefore, biomaterials are divided into four main classes: metals, ceramics, polymers, and composites, and are aggregated in different generations (Figure 3) of materials, according to their structures (traditional and emerging), functionalities, and/or response to the biological environment (bioinert, bioabsorbable, and bioactive).^28−32^

Historically, the development of bone biomaterials or first-generation biomaterials began in the 1960s and throughout the 1980s, as inert ceramic (Al_2_O_3_ and ZrO_2_) or metallic substitutes (stainless steel, titanium, or cobalt-chromium alloys), having wide applications in orthopedics, with the purpose of mechanical support at the site of the bone defect, such as larger load-bearing implants (femoral head, hip balls, and also substitutes for total joint and knee prostheses), or small components, such as pins, screws, and plates. A wide variety of polymers including polymethyl methacrylate, polyaryletherketones, and ultrahigh molecular weight polyethylene have also been evaluated as a bone substitute.^33^ However, only a few of them are suitable to be used as unique constituents of a final implant. Therefore, the first generation of biomaterials was mainly focused on bioinertia, where the focus was not to provoke a foreign body reaction in the organism.^34^

The second generation of biomaterials aimed to achieve bioactivity, which resulted in a further development of resorbable biomaterials, with controlled reactions with application regions and controlled release of drugs.^34^ It includes the evolution of bioceramics and biomaterials with a metallic or polymeric base over the years, as they have undergone modifications to form composites with a mixture with other materials (carbon nanotubes, HAp, graphene oxide). It occurred in parallel with the evolution of materials science, surface chemistry, and biological/clinical evidence, through reinforcement procedures, to improving mechanical and chemical properties and new applications of the biomaterials. In the second generation, the development of so-called bioactive scaffolds was also performed, with a composition similar to bone and porous structures, which is capable of promoting the formation and osseointegration of new bone (direct connection between living bone tissue and the surface of an implant).^33^

The original function of scaffolds is to provide adhesion substrates for cells, which must serve as inert physical supports. However, with the advancement in research on these structures, it is widely accepted that these materials can be active for cells, because they strongly regulate cell growth and differentiation due to their chemical, structural, and mechanical properties and, finally, contribute to the regeneration of the tissue.^35−37^

The developing field of tissue engineering aims to regenerate damaged tissues by combining cells of the body with highly porous scaffold biomaterials, which act as templates for tissue regeneration, to guide the growth of new tissue.^38^ Therefore, the third generation of biomaterials capable of regenerating functional tissues, such as interactive for functionality, integration, proliferation, differentiation for growth, or healing processes.^34^

In the fourth generation, there are emerging biomaterials or intelligent biomaterials that are composed of intelligent, customizable, and biologically active biodevices, that is, endowed with multiple biofunctionalities that combine therapy and regeneration, and therefore are capable of promoting bone and tissue regeneration while trying to balance metabolism along with treating concomitant pathologies of cellular compromise and divergent infections.^30,33^

In this group, there is a growing interest in the effect of electric fields on cells, with recent bioelectricity-based approaches to modulate cell fate by delivering bioelectric signals using electrophysiology or by activating electrical charges, induced by the inherent chemistry and nanostructure of biomaterials, such as the doping of specific ions in the structure of the HAp phase, which increases the segregation of several ionic species on the surface, which is responsible for increasing the osteogenic and antibacterial capacity.^33^

Therefore, studies in this field aimed at the development of biodegradable materials based on calcium phosphates, in which they have advantages ranging from good compatibility with the host tissue to biomimetic properties,^39,40,41^ and are discussed in the present review.

### 2.1 Hydroxyapatite: Structure and Methods of Preparation

HAp is a calcium phosphate from the apatite group and is the most stable phase at neutral or basic pH.^42^ HAp can occur in monoclinic or hexagonal forms (space group *P*63*/m*, *a* = *b* = 9.42 Å, *c* = 6.88 Å, ICDD 00-09-0432), although the hexagonal structure is more important in practical applications. Ionic substitutions in the HAp structure can decrease the symmetry, so that the unit cell is slightly distorted from the hexagonal structure.^42-47^

**Figure 4:**
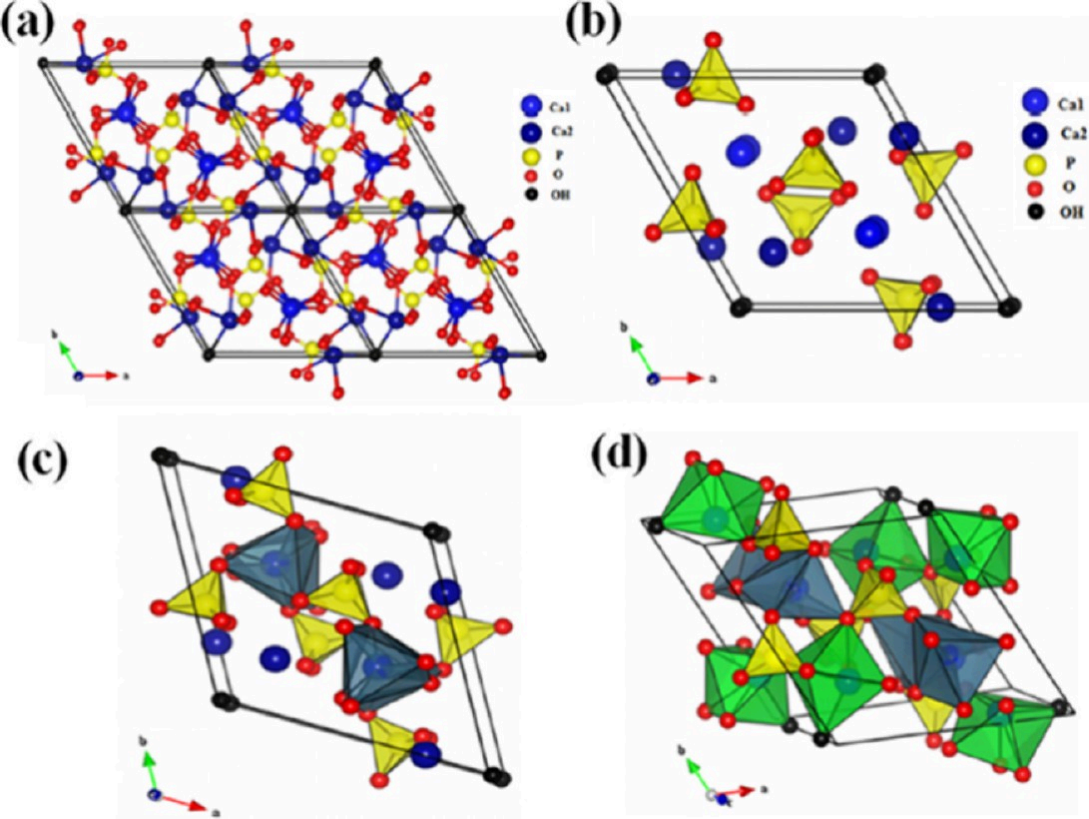
(a) Projection of the unit cell of HAp according to plan (001); (b) projection showing the arrangement of the octahedrons [Ca(1)O_6_] in the HAp structure; (c) projection showing the sequence of octahedral [Ca(1)O_6_] and tetrahedral [PO_4_] in the HAp structure; and (d) projection showing the sequence of octahedral: [Ca(1)O_6_] and [Ca(2)O_6_], and also tetrahedral [PO_4_] in the structure of HAp. Reprinted from Fihri et al. ref (43). Copyright 2017, with permission from Elsevier.

Structurally, stoichiometric HAp (1.67 Ca/P) is composed of a network of PO_4_^3–^ ions, forming channels that are filled with calcium ions, which occur at two different sites (I and II sites), Figure 4. Ca^2+^ ions play an important role in the properties of apatites.^43,44,48^

The Ca^2+^ ions at site (I) are located on the outside the channels and have strong interactions with phosphates, so that changes in this site compromise the entire crystalline network of HAp. On the other hand, the Ca^2+^ ions at site (II) are in the inner walls of the phosphate channels and allow modifications without compromising the structure.^43,48,49^

The channels still host the hydroxyl groups, which are present in perpendicular columns in the center of the channels and surrounded by Ca^2+^(II) ions, along the *c*-axis to balance the positive charge of the matrix. The channel diameter confers a certain mobility to OH^–^ ions and, consequently, allows their movement along the channels in the direction of the *z* axis.^42,43,48^

HAp also allows substitutions in all ions (cationic and/or anionic substitutions), and thus modifications in specific sites of interest can be modulated, without interference in the formed phase.^43,47,49^

**Figure 5:**
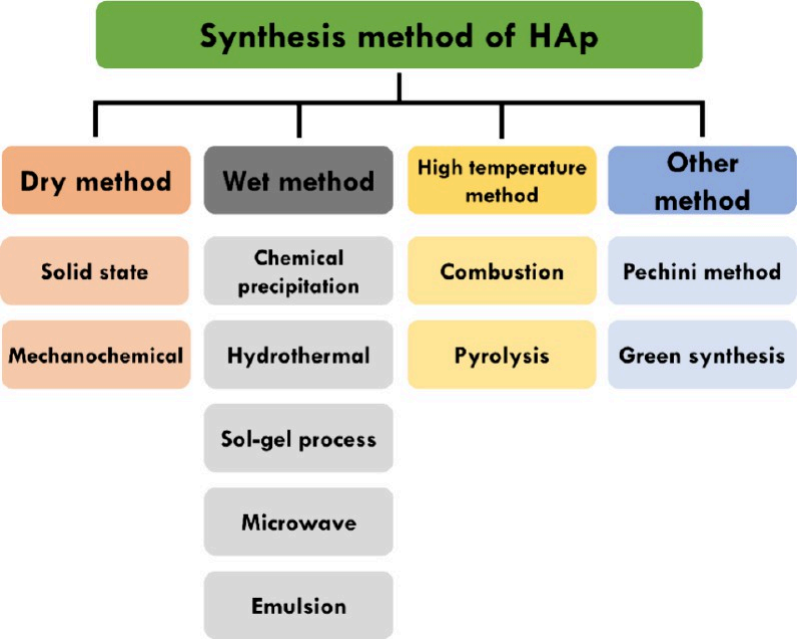
Examples of methods for obtaining HAp.

HAp can have natural or synthetic origins, in which preparation involves several methods. A brief summary of these methods is presented in Figure 5. The many ways the synthesis of HAp can been classified are as dry, wet, and high-temperature methods and also combined synthesis.^43,50,51^

**Figure 6:**
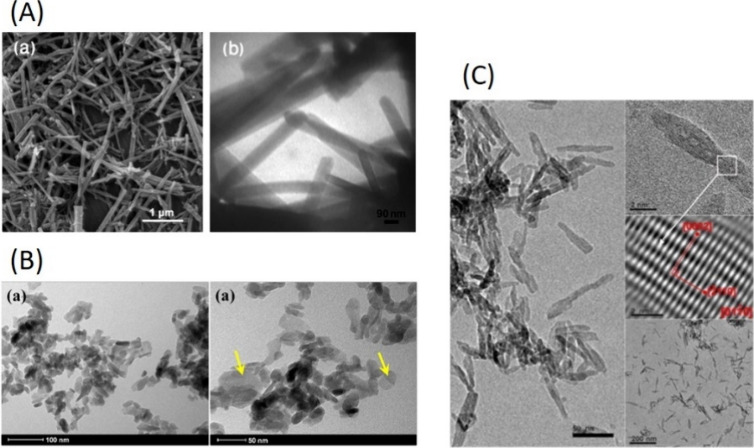
(A) (a) SEM and (b) TEM images of the HAp nanorods. Reprinted from ref (58). Copyright 2011, with permission from Brazilian Chemistry Society. (B) TEM image of (a) HAp. Reprinted from De Lima et al., ref (54). Copyright 2021, with permission from Elsevier. (C) TEM micrograph and SAED of the dried HAp, aged for 24 h in air. Reprinted from Bacan et al., ref (56). Copyright 2013, with permission from Elsevier.

Therefore, HAp presents different characteristics like morphology and crystal size as a function of the preparation and precursors chemicals used in each method. The method also influences the yield of different crystalline phases of calcium phosphate besides pure crystalline HAp.^50^ Indeed, the bioactivity, mechanical, and biological properties of HAp are significantly modified depending of the synthesis, which allows the modulation of the HAp for the desired application.^13,17,43,52,53^ Typical morphologies include hexagonal shape,^45,54^ rod-like morphology,^55,56,57^ and nanorod,^58^ and some examples are given in Figure 6.

**Table 2:** Main Methods for Synthesis of the HAp

Method		Advantages	Disadvantages	Reference
Dry methods	Solid state	Solvent is not necessary.	High temperatures for sintering and the presence of secondary phases	(59)
	Mechanochemical	Compression, shearing, or friction through grinding to induce chemical transformation	Irregular particle size	(13)
Wet methods	Chemical precipitation	Easy procedure, use of low temperatures, and high purity HAp	Particle agglomeration	(60)
	Hydrothermal method	Produces highly crystalline materials in one step	Use of higher temperatures and pression	(61)
	Sol-gel	Use of lower temperatures than conventional routes and formation of a highly pure product	High cost, use of alkoxide as a precursor, strict process control, and longtime reaction	(62)
	Emulsion	Highly pure HAp	Use of specific solvents and surfactants	(63)
	Microwave	High yield, crystalline solid, and homogeneous in size, porosity, and particle morphology, brief time reaction	Energy consumption	(43)
High temperature methods	Combustion	Rapid reactions, high- and purified crystalline solid, single-step, accessible precursors, and low aggregation particles	Energy consumption	(63)
Other methods	Pirolysis	High crystallinity HAp	Energy consumption	(43)
	Pechini method	Control the stoichiometry of the samples		(64)
	High-temperature organometallic injection with Ca and P precursors	Control of calcium phosphate nucleation-growth kinetics. Possibility of synthesizing monodisperse nanorods and nanowires with diameter <10 nm, length <200 nm and controllable length-to-width ratio.	Required application of high temperatures, high cost, and toxicity of the chemical precursors	(65)
Green synthesis	Use of nontoxic chemicals by using available natural resources. HAp obtained from natural resources has better osteoconductivity than HAp produced from synthetic compounds.	High risk of infection and disease transmission depending of the natural sources		(52)

In the dry method, precursors are accurately weighted and homogeneously mixed without the addition of solvent, and then the sintering process is performed to produce the porous solid. A major disadvantage of this method is the high temperature requirement (1250 °C).^43,63^ In some syntheses, the precursors are mixed for a long time (16 h) in a mill until a paste is formed, which is dried, for example, at 80 °C to form a powder which is then cold pressed. After this, the solid is placed in an oven at 500–1250 °C, followed by cooling.^63^

HAp can also be obtained by wet methods, such as chemical precipitation,^66,67^ sol-gel process,^68^ a hydrothermal method over conventional^69^ or microwave heating^70^ and emulsion^71^. These methods result in less agglomerated products and require simple experimental procedures.^43^ Chemical precipitation is one of the most widely used methods for synthesis of HAp in which water or another solvent can be used.^13,43^

Synthesis by chemical precipitation depends on the mechanism of aggregation-agglomeration-growth of HAp particles and the formation of nontoxic by-products, such as water, in addition to the use of low temperatures during the reaction (<100 °C), which are the advantages.^72^

In chemical precipitation, the procedure involves the preparation of precursor solutions (calcium and phosphate salt solutions) for the formation of apatite, followed by the steps of precipitation, aging, or maturation of the precipitate, filtration, and drying. The control of parameters during the process, such as the reaction temperature, the rate of addition of precursor solutions, ion concentration, and drying conditions, is decisive in the shape, particle size, and stoichiometry of the synthesized solids so that the control of these parameters allows obtaining stable materials, ensuring that these solids have characteristics close to those of natural HAp.^13,43,63,72^

The synthesis of HAp by the emulsion method involves the use of precursors, specific solvents, and a surfactant, in addition to the control of pH and temperature. There are reports of obtaining high purity HAp by adding cetyltrimethylammonium bromide, cyclohexane, and alcohol to calcium and phosphate precursors under vigorous agitation (1800 rpm) and subsequent calcination at 300–850 °C.^73^

Methodologies involving the use of high temperatures for the synthesis of HAp require a high energy consumption, including the use of microwaves. The preparation of HAp using high temperatures results in a better yield, in addition to being a crystalline and homogeneous solid in size, porosity, and particle morphology. The advantage of using microwaves is due to two factors: i) the molecular agitation is purely thermal, caused by the inversion of the dipole with the extremely fast alternations of the electric field, and ii) it is of an electrostatic origin, involving interactions such as dipole-dipole interactions between polar molecules and the electric field. This directly affects the reaction kinetics, allowing for a decrease in the activation energy.^43,73,74^

An alternative way to synthesize HAp is by using the Pechini method, which has gained prominence compared to conventional methods.^75^ Variables such as temperature ranges and proportions of citric acid and metallic cations can be changed, allowing the control of the stoichiometry of the samples. In this method, there are better kinetics of crystallization and particle growth through the use of ethylene glycol as a solvent for the polymerization process between citric acid and the metallic cations involved.^75^

The Pechini method consists of the formation of a chelate between the metal element and an α-hydroxycarboxylic acid in the presence of a polyhydroxy alcohol that the polyesterification reaction under heating forms a resin.^76^ The Pechini method was first proposed for the synthesis of alkaline earth titanates, zirconates, and niobates by reacting 1 mol of the metal precursor (hydrated oxides, alkoxides, hydroxides, and carbonates) and 2–8 mol of citric acid in excess of ethylene glycol. Over time, the method was extended to several other materials using other precursors such as nitrates and acetates. In addition, other synthesis steps have been used, such as pH control, grinding of the polymeric precursor, and calcination in an oxidizing atmosphere to eliminate excess carbon and reduce the formation of agglomerates.^76^

HAp can be obtained by other methods involving high temperatures, such as the combustion of precursors during a certain period of time. The final products are pulverized by milling to obtain homogeneous hydroxyapatite particles.^63^

The synthesis of HAp by combustion results in a high purified solid, and the reaction occurs in a single step, by combining accessible precursors and simple procedures. In general, the synthesis of HAp by combustion involves a very rapid exothermic and self-sustaining redox reaction between oxidizing species (calcium nitrate and nitric acid) and an organic fuel (e.g. urea, glycine, ammonium acetate, ammonium citrate, citric acid, hydrazine, and malonic dihydrazide), which are prepared in an aqueous solution.^13^ Other precursors are used for the formation of HAp, including Ca(NO_3_)_2_, and are (NH_4_)_2_(HPO_4_) and subsequently mixed, followed by the addition of concentrated nitric acid, as a way of dissolving the formed precipitate; one or more fuels are incorporated in the resulting solution. After this, the reaction will start by heating the mixture in an oven to an initial temperature (i.e. 300 °C), with a sudden increase in temperature, as a result of combustion, up to a maximum value that depends on the fuel. In fact, different fuels provide different flame temperatures ranging from 100 to 900 °C (e.g. citric acid: 150 °C; succinic acid: 425 °C; urea: 800 °C; glycine: 890 °C). The cooling of the mixture should be fast as a way to induce maximum nucleation and avoid disordered particle growth. The exothermic nature of the combustion reaction provides the necessary heat to maintain the temperature of the system, and, once started, external heating is no longer necessary. Several parameters during the synthesis, such as, for example, the fuel used as an oxidizer, the initial temperature of the furnace, the nature of the fuel, and the amount of the initial precursor, influence the maximum reaction temperature (i.e. the temperature of the flame) and affect the characteristics of the solid obtained.^13^

The high temperature organometallic approach can be an alternative option, which was successful in obtaining a variety of high-quality monodisperse nanoparticles. This approach relies on the appropriate precursor and growth condition so that nanoparticle can nucleate from homogeneous solution, and its nucleation-growth kinetic can be finely tuned. However, such an approach is difficult to perform for calcium phosphate nanomaterials due to the lack of appropriate phosphate precursors.^65^

Another way of obtaining HAp is the green synthesis method in which natural materials are used that allow the replacement of calcium and phosphate precursors.^77^ Natural resources can be biological waste, such as egg shells, fish bones, and bovine bones, in addition to the calcination of organic matter such as shells, corals, starfish, and algae.^13,43,63,77^

A more detailed explanation of the preparation methods of the HAp was extensively reported.^13^

### 2.2 Polysaccharides

**Figure 7:**
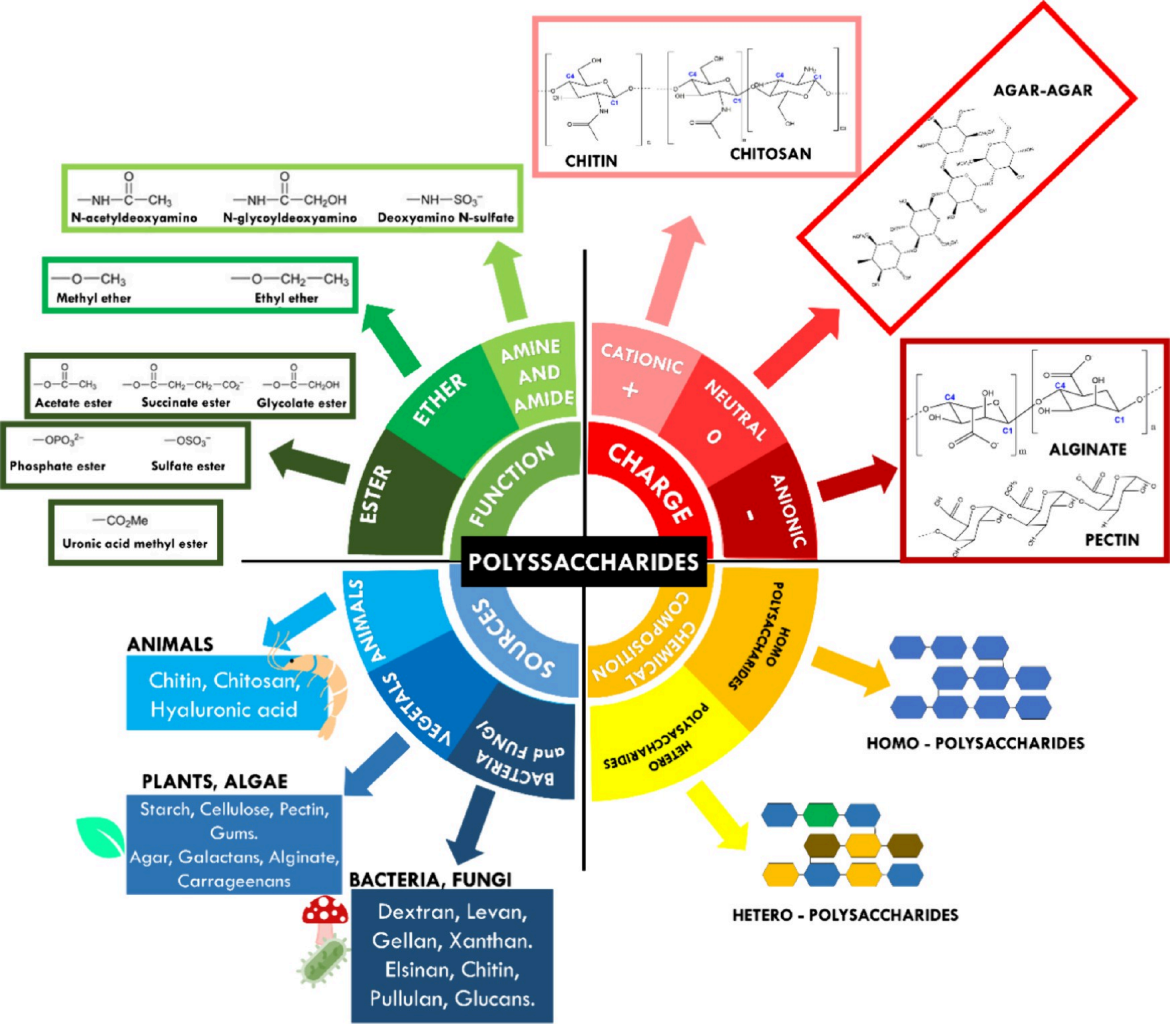
Classification of polysaccharides in relation to their function, charge, source, and chemical structure.

Polysaccharides are carbohydrate polymers composed of several smaller monosaccharide units linked by glycosidic bonds or covalently bonded to other molecules including amino acids, lipids, and peptides.^78,79^ The arrangement of the units of monosaccharides in polysaccharides can present a repeating homo or hetero and, depending on which monosaccharides are connected, which carbons in the monosaccharides connects, polysaccharides take on a variety of forms, including linear or branched structures (Figure 7). They are the most abundant compounds in nature among carbohydrates, and their origins include natural sources such as plants and also subproducts from industrial processes.^78,79^

Examples of polysaccharides obtained from natural sources are cellulose, starch, chitin, chitosan, and alginate,^80^ whose complex secondary structures perform several functions in plants, animals, and different other microorganisms. Their properties including hydrophilicity, good stability, safety, lack of toxicity, and biodegradability in nature make some of them good candidates for food packaging, pharmaceutical formulations, and various kinds of sustainable and renewable products in biomedical industries.^79^ Furthermore, their low cost, easy and bulk availability, antimicrobial properties, adsorption characteristics, and high porosity are desired properties for their wide applications.^81^

Depending on the structure, polysaccharides can be classified based on the polyelectrolyte charges such as negatively charged polysaccharides (e.g., alginate, heparin, hyaluronic acid, and pectin) and positively charged polysaccharides (e.g., chitin, chitosan). Among them, this review focused on anionic alginate and pectin types, besides chitosan, which is a cationic polysaccharide. They are selected considering their importance in the biomedical field in combination with biological calcium phosphate such as HAp.^82^

In fact, the importance of polysaccharides in the biomedical field is due to their properties. Reactive side chain groups of polysaccharides are advantageous for functionalization with nanoparticle-based conjugates or therapeutic agents such as small molecules, proteins, peptides, and nucleic acids. Polysaccharides show excellent pharmacokinetic and drug delivery properties, facilitate improved oral absorption, control drug release, increase retention capacity *in vivo*, targeted delivery, and exert synergistic effects.^83^

#### 2.2.1 Alginate

Alginates are unbranched polyanionic polysaccharides constituted by (1–4)-linked β-d-mannuronic acid (M unit) and α-l-guluronic acid (G unit), which are irregularly linked by β-1,4-glycosidic bonds. Different proportions of G and M blocks are possible and influence the alginate properties, while the length of connected G units is directly related to mechanical properties, and the content of M units determines immunogenicity.^79,84,85^

A natural alginate can be obtained from seaweed and some bacteria, and sodium salts of the alginic acid are generally a common form. They are widely distributed in cell walls and intercellular mucilage of different species of brown algae such as edible ones (*Phaeophyceae*), as well as *Laminaria hyperborea*, *Laminaria digitata*, *Laminaria japonica*, *Ascophyllum nodosum*, and *Macrocystis pyrifera* and also some bacteria such as Pseudomonas and nitrogen-fixing bacteria.^79,85^

The alginate properties include different gel strengths, hydration capacity, viscosity, and bioactivity that are associated with their differences in molecular length, M/G residue ratio, and distribution, as well as the degree of acetylation, favorable biocompatibility, biodegradability, solubility, stability, and specific physiological functions such as hypoglycemia, antioxidation, and enhancing immune activity.^85,86^

The presence of hydrophilic groups such as hydroxyl and carboxyl groups favors the chemical or physical properties of the alginates, improving their applications in medicine, cosmetics, environment for water treatment and foods ,as well in the biomedical field, where they are used for soft tissue regeneration as 3D hydrogels and 2D membranes.^86^

#### 2.2.2 Chitosan

Chitosan is a cationic copolymer composed of *N*-acetylglucosamine and glucosamine units linked by ((1,4)-2-amino-2-deoxy-beta-d-glucan), which can be obtained from the deacetylation of chitin. Chitin ((1,4)-*N*-acetil-d-glucos-2-amine) is a naturally abundant polysaccharide found in shellfish, marine organisms, fungi, and insects.^87,88^ Acetylation of the chitin transforms its amide group into an amino group that forms chitosan, and the percentage of amide groups converted an into amino groups is defined as the degree of deacetylation (DD). DD affects the properties of chitosan, including its solubility in aqueous acetic solutions and reactivity.^87,88^

The presence of different functional groups in the chain of chitosan such as amino, amide, hydroxyl, and carboxyl groups results in possible interactions with substances by chemical modification and grafting to the production of new materials desired for specific applications.^88^ Amine groups in chitosan can undergo extensive protonation in aqueous media, allowing electrostatic interaction with solvent molecules, additives, or biological sites. This property enables both physicochemical modification and the preparation of composites, including nanocomposites. Furthermore, because of their nontoxicity, biocompatibility, and biodegradability, materials containing chitosan are widely used in medicine, and tissue engineering, and many drugs with antiallergic, anticancer, antiviral, and antimicrobial activities have been developed on chitosan.^10,88,89^

#### 2.2.3 Pectin

Pectins are anionic heteropolysaccharides that belong to a group of plant-derived complex carbohydrates composed of several units of hydrogalacturonic acid units (β-(1-4)-linked-d-galacturonic acid with galactose and rhamnose).^90,91^ This complex mixture of polysaccharides enriched with galacturonic acid units (made up of various sugars such as galactose, rhamnose, arabinose, xylose, and glucose) and galacturonic acid methyl ester, which make up about one-third of the dry matter of plant cell walls, i.e. form the chemically and physically stable tissues of plants, when combined with proteins and other polysaccharides.^92^ Pectin occurs in cell walls (up to 30%) of mono and is dicotyledonous plants, and biosynthesized in Golgi vesicles of plant cells along with fibrins. Pectin has direct action to hold plant cells in unity, providing strength and elasticity to the cell wall, and protect the plant from harsh environmental conditions including low temperatures and acidity.^91^

Pectin is named for water-soluble pectinic acids with varying degrees of neutralization and methyl ester content and are capable of forming gels with sugars and acids under appropriate conditions. Therefore, the classification of pectin is made in function of the methoxyl content in low methoxyl pectin (<50% esterification) or high methoxyl pectin (>50% esterification). High methoxyl content and are more commonly used types, which is responsible for the low ability for gelling. On the other hand, the low methoxy pectin requires a relatively high sugar and acid content to form a gel, although gelling ability can be modified in the presence of certain sugar-free metal ions.^90^

Commercial pectin is obtained from a few plants, including citrus peels, rose hips, and apples, especially as a by-product in the manufacturing of the juice industry such as in citrus and apple juice.^91^

Pectin can be represented as a single extracellular matrix, which is a complex structure that is continuously formed throughout the plant body. The chemical structure and properties of pectin, i.e., molecular weight, viscosity, gelling, and/or emulsifying ability, are dependent on their origin (type of plant and the section of the plant), as well as the extraction method and environmental factors. Pectin is easily available, biocompatible, biodegradable, non-toxic, and good for human health because their hypoglycemic and hypocholesterolemic properties, its use in various pharmaceutical and biomedical applications, and its success in the food and beverage industries as a thickening agent, gelling agent, and colloidal stabilizer.^92−94^ However, some limitations of its use in drug delivery devices and tissue engineering scaffolds are the low mechanical strength and rapid degradation rate.^91,95^ However, some alternatives to improve and change the properties of pectin are modifications or combinations with other molecules or organic polymers and/or inorganic compounds. In fact, the presence of functional groups hydroxyl, carboxyl, carbomethoxy, and acylamino makes pectin capable of obtaining a wide variety of derivatives and applications. Modifications also include the production of composites or pectin blends such as hydrogels, sponges, membranes, or 3D printed matrices for different applications in the biomedical field.^91,95^

### 2.3 Polysacharides Hydroxyapatite (Nano)composites

The human body is unable to regenerate extensive defects because of the lack of an extracellular matrix to fill these defects, although bone tissue is formed by a continuous organic phase reinforced with nonstoichiometric HAp crystals. The natural calcium phosphates have disadvantages, such as small crystals and low crystallinity, in addition to significant amounts of foreign ions and poor mechanical properties, which are limitations that need to be overcome for biomedicine applications.^96−98^

However, the polysaccharides contain the ability to mimic the microenvironment of the natural extracellular matrix. Therefore, they feature a 3D biological environment, which is ideal for regeneration, since the extracellular matrix plays an important role in the restructuring of damaged tissue, like, for example, providing physical support to cells, and also regulating cellular activity that includes growth, proliferation, differentiation, migration, homeostasis, and morphogenesis.^99−101^ Therefore, these properties allow the human body to regenerate small defects in skin, cells, organs, and biological or bone tissues.^99−101^

In this sense, biomimetic materials based on ordered deposition of HAp crystals in systems with polysaccharides can have the potential to regenerate bone tissue.^97,102^ Systems based on HAp and polysaccharides are materials with a controlled morphology and have similarity to natural bone tissue, and act in the damaged tissues, inducing, for example, the integration of the graft with the host tissue, resulting in a fully functional tissue.^97,102^

The structural nature of bones inspired scientists to develop nanostructured biocomposites from different types of materials of natural or synthetic origin. Therefore, HAp @polysaccharides hybrids showed significant influence on biocompatibility and functional performance, for example, in bone tissue engineering.^97,103^ Furthermore, the interaction between the components of these biocomposites with structures in nanometric dimensions and tissues or biological materials made nanotechnology an attractive area for biomedical applications.^103^

Among these nanostructured biocomposites, the polysaccharide@HAp nanocomposites have different polysaccharides such as chitosan,^21,97,104^ alginate,^105−107^ and pectin,^23,108^ among others. They have advantages over other synthetic materials, such as adjustable mechanical properties, biocompatibility, biodegradability, and even antibacterial activity.^97^

In this context, polysaccharides@HAp bionanocomposites have been a promising target of study. Research carried out obtaining nanostructured systems through the incorporation of polysaccharides in apatite for surface engineering has indicated it is a significant strategy, since it allows changing the structure of the material or incorporating specific binders to overcome the mechanical deficiencies of HAp-based biomaterials, enabling the adaptation of specific requirements to overcome the main therapeutic barriers in several diseases.^109,110^ Apatite polysaccharide nanostructured systems achieve characteristics such as controlled size and distribution, suitable surface functional groups, and prespecified properties to be applied to desired targets, such as controlled drug release capabilities to treat specific diseases.^109,110^

Organic@inorganic hybrids are widely studied for drug release, for developing an ideal system that can guarantee controlled drug release during the first hours, followed by a release above the minimum inhibitory concentration, which must be maintained for a longer period (days to weeks).^111^

Therefore, this review focused in bionanocomposites based on HAp and natural polysaccharides like alginate, chitosan, and pectin for biomedical applications, including vehicles for drugs, bone implants, and others.

#### 2.3.1 Synthesis and Characterizations

Biomaterials produced from calcium phosphates have been widely used as artificial bone grafts, since they can mediate bone integration through the stimulation of osteoclasts or osteoinduction capacity, which concerns cellular regulation.^112^ Cellular regulation is a frequent and essential phenomenon for bone consolidation or healing, which involves the recruitment and stimulation of osteoblast cells to develop into bone tissue.^112^

For bone induction to be efficient, the calcium phosphate must have specific pore sizes and particle sizes. Therefore, to use calcium phosphate in the treatment of diseases related to the bone system, it is necessary to control some aspects such as the chemical elements present in the composition, morphology, and particle size of the material.^112,113^

In an attempt to establish a balance between the mechanical and structural characteristics and essential constituents of biological tissues for the treatment or correction of bone defects, biocomposites manufactured from natural polymers are a possibility for application in tissue engineering, for example, as the production of grafts to improve the restoration process of host bone tissue.^80,114^

Over the years, different routes for obtaining of nanostructured polysaccharide@HAp systems have been developed to achieve specific properties and characteristics for different applications. In this sense, some of this research is reported here, emphasizing the synthesis and surface characteristics, such as the size and distribution of the particles, and the functional groups of the composite systems in focus: alginate@HAp,^103−107^ chitosan@HAp,^115−117^ and pectin@HAp.^118^

Therefore, the use of various natural and synthetic polymers as templates for the growth of HAp allowed the production of materials that will serve as a support for osteogenic activity. In this way, HAp and bioactive molecules, such as polysaccharides and proteins, are possible in the preparation of biomaterials, as these bioactive molecules will function as an organic template during the preparation of these different structures that help in the repair and construction of bone tissue.^119^

**Figure 8:**
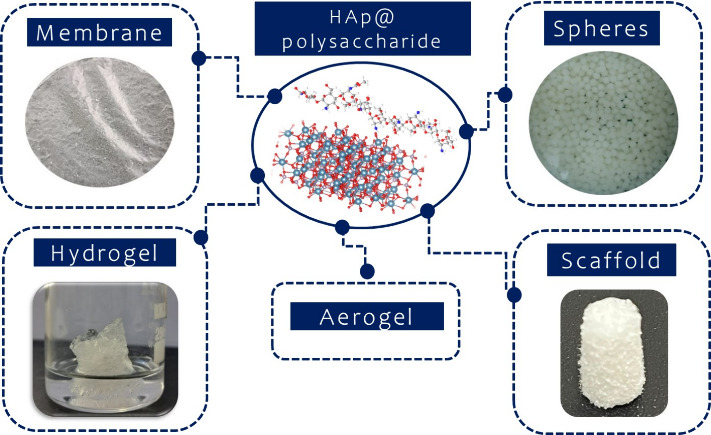
Different types of structures for polysaccharide@HAp nanocomposites.

Several types of structures have been designed and studied to develop materials with desired properties that combine both the ability to promote bone healing and accelerate the release of immobilized cells or active ingredients that can prevent infection processes through controlled drug delivery. Among the materials that can be highlighted a) membrane,^120^ b) hydrogel,^121^ c) scaffolds,^122^ d) aerogels^78^ and e) spheres are summarized in Figure 8.

Hydrogel structures based on polyvinyl alcohol, gelatin, sodium alginate, and nano-HAp were obtained and evaluated *in vitro* at 37 °C in the release of aspirin. The nanometric size, morphology, and crystalline state of the nano-HAp particles allowed the hydrogel the ability to absorb and bind drug molecules without altering the pharmacokinetic structure. Therefore, the results indicated that the incorporation of nano-HAp can significantly improve the hydrogel structure, allowing its use in drug delivery systems.^123^

Generally, the synthesis for nanocomposites based on chitosan@HAp followed the same methodology, only varying the way in which these materials are obtained, like in spheres,^124^ scaffolds,^122^ or hydrogels,^125^ which is dependent on the crosslinking agent. One of the first hybrid materials of chitosan@HAp with a homogeneous dispersion of the phases was obtained,^124^ and the influence of organic acids (acetic acid, lactic acid, malic acid, and citric acid) was evaluated in the formation of the chitosan@HAp composite.^124^ Other different methodologies were used to prepare scaffolds based commercial HAp and chitosan for vancomycin adsorption.^126^

In a new method for the acquisition of scaffolds, chitosan was dissolved in a dilute acetic acid solution under continuous stirring, and acrylic acid and *N*,*N*-methylene-bis-acrylamide were added as monomer and cross-linking agent, respectively.^122^ Then, HAp was introduced into the reaction forming a hydrogel. Different nanocomposites containing 1:0.25, 1:0.5, 1:0.75, and 1:1 chitosan@HAp were synthesized and used to produce the scaffolds. The loading of anti-inflammatory naproxen on the scaffolds was higher with increasing HAp content, which can be attributed to the increase in porosity that allowed better interaction between the drug and calcium phosphate.

The hydrogel based on alginate microparticles and core–shell microspheres alginate@chitosan were synthesized in the presence of drug-doped HAp.^127^ To obtain these hydrogels containing propranolol hydrochloride and sodium salt monohydrate, a method was carried out based on the use of a microfluidic system to obtain a crosslinked microparticulate hydrogel with homogeneous sizes and morphologies, integrating external and internal gelation. The microparticulate hydrogel exhibited a spherical core–shell structure, with the presence of a fibrous surface that will play a major role diminishing the hydrogel degradation and modulating the delivery of drugs.

Pectin has several properties making it a promising and widely used material for various applications such as pharmaceuticals, food packaging, and cosmetics and can also be used as a matrix for the entrapment and/or delivery of a variety of drugs, proteins, and cells.^92,94^ Many of these studies focused on evaluating the influence of pectin on the structure of HAp-based materials.^128^

A pectin-based hydrogel and cellulose nanocrystals were used for nucleation and growth of HAp by the biomimetic method.^129^ The direct impact of different percentages of nanocrystalline cellulose in the pectin hydrogel and the influence of HAp obtained through two methods were evaluated. The cellulose nanocrystals were chemically functionalized using anhydrous maleic acid to incorporate vinyl groups.

In searches carried out on the Web of Science platform with the words: hydroxyapatite or HAp and pectin, the study developed by Iviglia et al.^115^ described a pectin@HAp hybrid for biomedical application. In this study,^115^ a porous scaffold was obtained by reacting HAp, β-tricalcium phosphate (β-TCP), and a polyelectrolyte system composed of pectin and chitosan for the transport of the antibiotic vancomycin.

Membranes developed for bone regeneration must have a suitable mechanical structure and chemical composition to mimic biological structures. In this sense, membranes with bilayers inspired by the periosteum were obtained through the crosslinking of alginate with different amounts of nano-HAp.^130^ The ionic interaction between alginate and nano-HAp improved the strength and microstructure of the hydrogels. Furthermore, distinct surface characteristics on each side of the membranes were obtained, resulting in a highly porous fibrous side and a mineral rich side with higher roughness and lower porosity. The amount of nano-HAp decreased the plasticity of the membranes and increased the degradation rate.^130^

**Figure 9:**
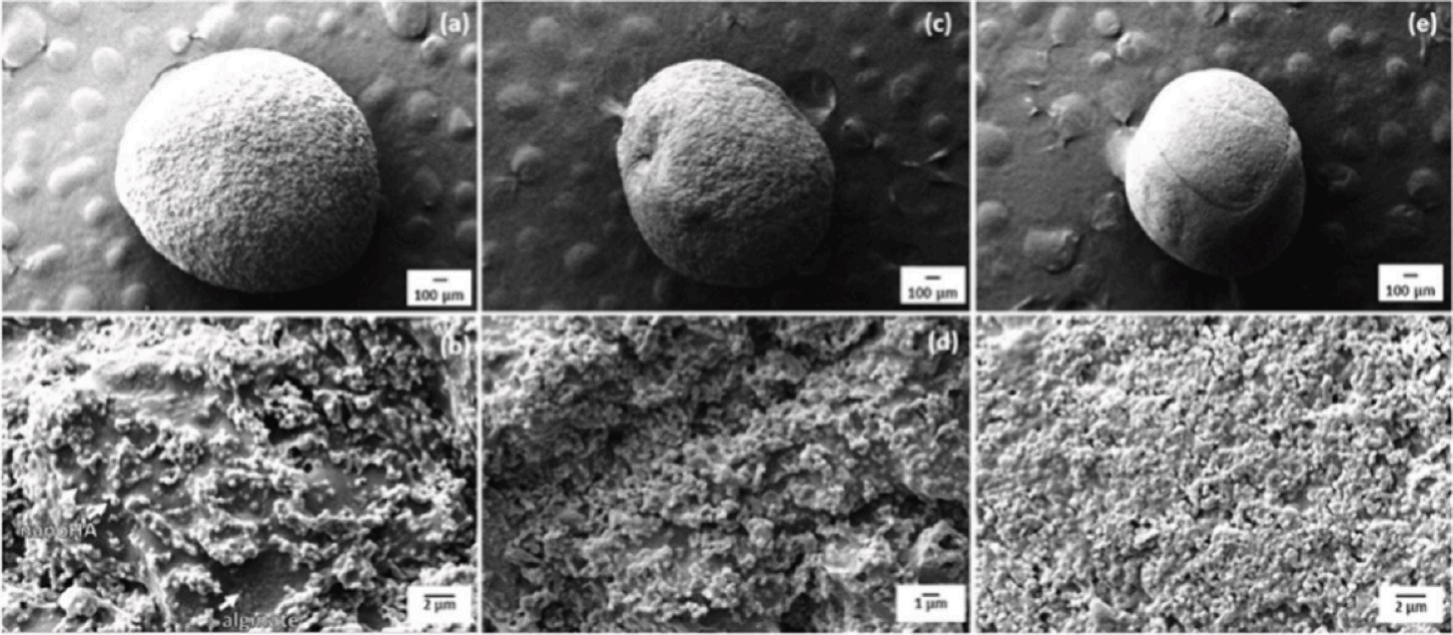
SEM images of the morphology and microstructure of alginate matrix particles containing HAp particles produced at caliber and magnification: 22G at (a) 100× and (b) 10,000× 30G at (c) 100× and (d) 10,000×, and 31G at (e) 100× and (f) 10,000×. Reprinted from Niero et al, ref (131). Copyright 2023, with permission from Elsevier.

HAp was obtained from poultry and shellfish by-products of nanometric size (61–72 nm) and high crystallinity (86–89%) and produced alginate spheres for use as biomaterials for tissue repair (Figure 9).^131^

The freeze-drying process allows for the opening of larger pores, even in very small sizes, which favors better fluid absorption. The most important reason for the high fluid absorption rate is related to the hydrophilicity of the alginate. The porosity of the optimized spheres was up to 90%, similar to that of human bone, and they did not show cytotoxicity. Therefore, the alginate@HAp spheres presented potential applications in tissue repair.^131^

Xu et al.^132^ reported the effects of adding silk fibroin to scaffolds produced using the 3D printing technique by pneumatic extrusion prepared with HAp and alginate, and evaluation of the mechanical properties. Reliable mechanical properties ensure the functionality and durability of scaffolds and play an important role in bone tissue engineering. Silk fibroin has stability, water insolubility, heat resistance, and mechanical properties. Therefore, the presence of polysaccharide in the alginate@HAp composite allowed the alternating hydrophilic and hydrophobic chains. According to the authors, hydrogen bonding between adjacent chain segments influenced the good tenacity and resistance of silk fibroin. The compressive strength test of the scaffolds resulted in 1 and 1.5 MPa, which is close to the minimum strength requirement of trabecular bone (1–12 MPa) and is very beneficial for cartilage and subchondral bone.^132^

An alginate@collagen@HAp composite structure containing porous HAp microspheres with amoxicillin was obtained through a three-dimensional printing model and freeze-drying.^20^ The morphology of the scaffolds indicated that they contained microporous structures and drug-loaded microspheres embedded in hydrogels with a diameter of 18.62 ± 2.77 μm. The mechanical properties of the scaffolds showed a 15% deformation, with an average compressive stress of 1.62 ± 0.09 MPa and an average compressive modulus of 11.28 ± 0.78 MPa. The swelling rate evaluated in each scaffold changed rapidly in the first 4 h and reached swelling equilibrium after 14 h. The swelling ratios of the composites at equilibrium varied between 800.84 and 948.50%. The results suggested that the addition of amoxicillin could increase the swelling rate of the composite scaffolds alginate@collagen@HAp.^20^

Bone tissue formation only occurs when a series of complex events take place, and the critical step is the mineralization of calcium phosphate in the extracellular matrix, which allows the formation of HAp crystals.^119^ Therefore, the presence of HAp in biomaterials for bone implants allows calcium and phosphate ions to be released simultaneously during the degradation process, favoring the bone regeneration process.^130^

The calcium phosphates most commonly used for the production of biomaterials are mainly HAp, *β*-TCP, and biphasic calcium phosphate. Crystallinity, porosity, structure, particle size, percentage of HAp and *β*-TCP in the biomaterials produced are one of several factors that affect the process of bone formation and development, that is, osteogenesis. To obtain excellent osteogenic effects, these materials are generally obtained by sintering at more than 1000 °C.^112,133^

Calcium phosphates are not induced by bone tissue to produce new tissue; however, calcium phosphates have the property of inducing bones to form new tissue, and this can happen in two ways: (i) the design and geometry of the scaffolds combined with appropriate phosphate porosity and (ii) growth factors are related to the bioactive molecules with which this phosphate will bind.^112,113^

**Figure 10:**
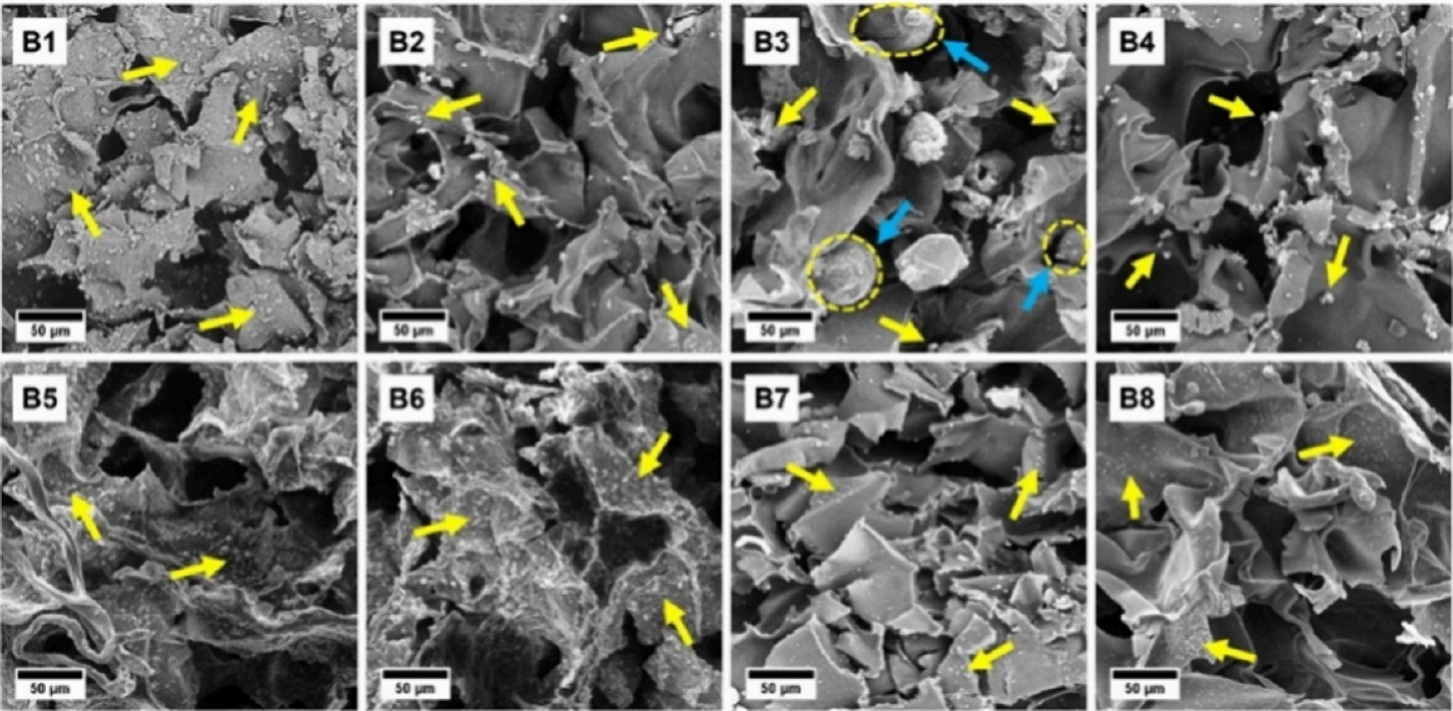
SEM micrographs of scaffolds (B4, B7, and B8) and scaffolds (B1–B3, B5, and B6). Yellow arrows indicate nano-HAp, and blue arrows indicate microspheres. Reprinted from Samie et al, ref (134). Copyright 2023, with permission from Elsevier.

Porous scaffolds with various compositions nano-HAp, chitosan, and hydroxypropylmethylcellulose or (*Bombyx mori*) silk fibroin were prepared by a freeze-drying method (Figure 10). Drugs and bioactives species were incorporated in the systems for application as a substitute for subchondral bone and provide dual functionality as local delivery of the payload into the extracellular environment and support for cells to organize themselves in a 3D arrangement.^134^

The images of the porous composite scaffolds showed an interconnected and stratified 3D porous structure with both micropores and macropores, with a mean pore diameter of the scaffold of 64 ± 1.06 μm, and the pore walls were found to have both smooth and rough surfaces due to the presence of the various polymeric/particulate components. The compressive mechanism of cancellous tissues is suitable for trabecular/spongy bone tissue and capable of releasing drugs/bioactives.^134^

Porosity is very important as the highly porous structure facilitates cell infiltration, which in turn compromises the mechanical properties of the scaffold. It was suggested that a scaffold for bone tissue regeneration should have a porosity >60%. Scaffold porosity is determined by solvent displacement; therefore, the overall porosity obtained from liquid infiltration remained <60% in all samples.^134^

A biodegradable chitosan@nano-HAp porous 3D scaffold was used in periodontal bone regeneration.^135^ The scaffold presented a fully interconnected porous microstructure (total porosity 78%, average pore size 200 μm), which is critical for bone regeneration, and resulted in the HAp formation in its surface after 21 days in simulated body fluid, demonstrating its bioactivity *in vitro*. Mechanical analysis indicated that the presence of nano-HAp significantly improved the storage modulus (42.34 ± 6.09 kPa at 10 Hz), suggesting that it can support bone growth in low-load bone defects.^135^

Critical bone defect is still an urgent problem in the field of bone repair, and in this sense a new type of structure using chitosan and HAp was obtained^22^ with the addition of freeze-dried platelet rich fibrin (L-PRF). The scaffolds presented compressive properties that decreased with the L-PRF content. The scaffold with the lower compressive strength and modulus presented values of (552.5 ± 21.5) kPa and (9.8 ± 1.9) MPa, respectively, and the scaffolds containing chitosan and HAp showed the higher compressive strength and modulus, up to (994.5 ± 82.9) kPa and (22.1 ± 4.5) MPa. All systems presented resistance of the cancellous bone due to the decrease in the HAp proportion. However, the structure of HAp was disturbed with the addition of L-PRF, although the resistance of cancellous bone can be reached, with a compressive modulus of around 2–20 MPa.^22^

Shellfish by-products and derivatives were applied as raw materials for the production of nanocrystalline calcium phosphate hybrids.^9^ Mussel shells were transformed into HAp through dissolution-precipitation at 45 °C, while chitosan from shrimp shells was introduced as a reinforcing biopolymer to produce chitosan@HAp composites. In the obtained materials (∼90% relative density) by increasing the polymer content by up to 10% by weight, the flexural strength of sintered pellets increased from ∼45 MPa to ∼57 MPa, while the hardness decreased from ∼1.1 GPa to ∼0.8 GPa, better addressing the mechanical properties of cortical bone. Furthermore, the chitosan@HAp composites were bioactive, demonstrating their potential use for bone tissue engineering applications.^9^

Natural and synthetic polysaccharides and nano-HAp formed a porous structure to mimic the component and microstructure of natural bone.^3^ Gelatin, chitosan, and polyvinyl alcohol were used to simulate the extracellular matrix, which exhibited adjustable pore size, porosity, swelling, pH, degradation, and mechanical resistance. After incorporation of nano-HAp particles, the scaffolds showed better compressive strength, adaptability to pH, better surface bioactivity, and biomimetic structure.

**Figure 11:**
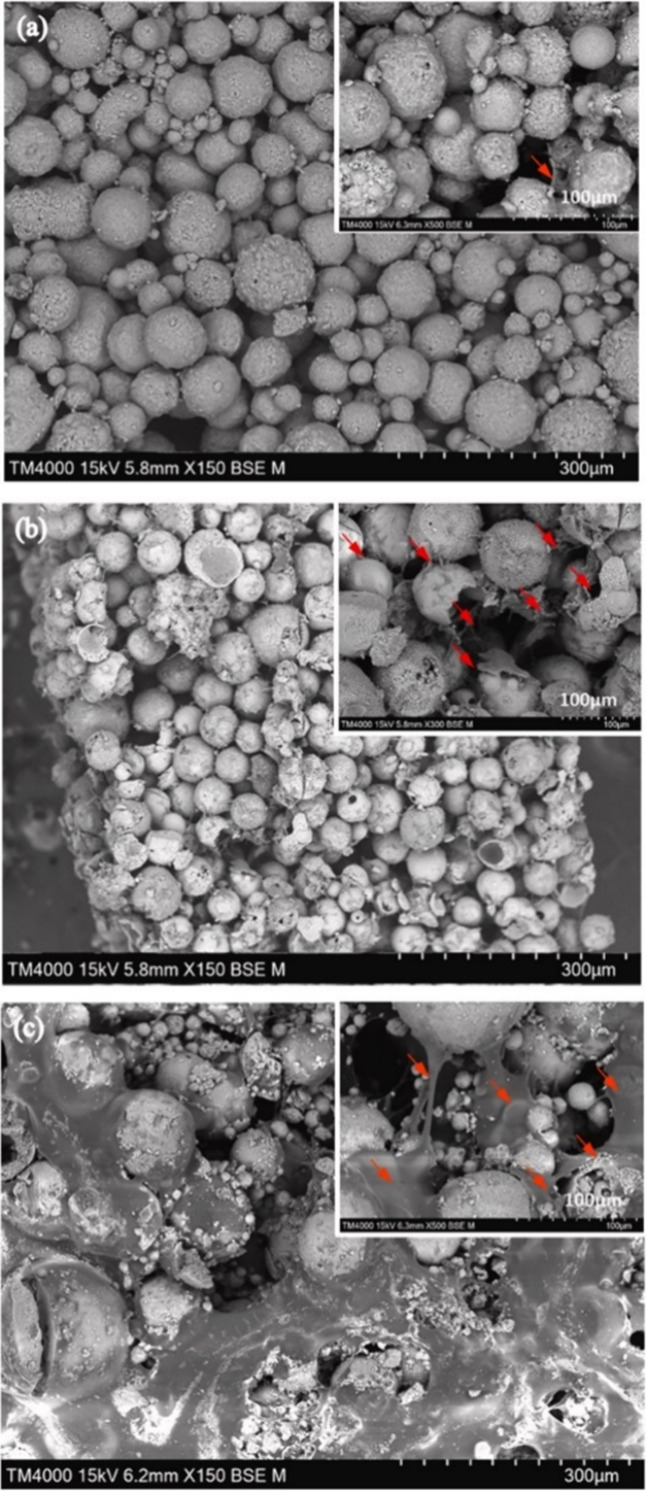
Morphologies of cross sections of samples printed with different component composite powders: (a) pure HAp sample; (b) carboxymethyl chitosan@HAp sample with 1 wt % carboxymethyl chitosan; (c) HAp@carboxymethyl chitosan sample with 3 wt % carboxymethyl chitosan. The red arrows show the connection between the HAp particles. (For interpretation of the references to color in this figure legend, the reader is referred to the Web version of this article.) Reprinted from Wei et al, ref (136). Copyright 2023, with permission from Elsevier.

The use of printed structures containing HAp to mimic bone structure is always a challenge in tissue engineering. Therefore, a series of carboxymethyl chitosan@HAp scaffolds were obtained by using 3D piezoelectric inkjet printing technology^136^ (Figure 11), and the quality of formation, structural morphology, mechanical properties, degradability, cytotoxicity, and cell adhesion growth were evaluated.

A higher carboxymethyl chitosan content decreased the quality of the samples and increased the pore size and porosity. However, when the carboxymethyl chitosan content reached 5% by mass, cracks appeared on the surface of the sample, and the quality of the composite was poor. The toughness of the composites was improved by incorporation of carboxymethyl chitosan in HAp, which was attributed to the stronger connections of the polysaccharide network between the HAp particles through hydrogen bonds.^136^

A new bone substitute called as biomicroconcretes presented high surgical practicality and satisfactory mechanical properties.^137^ The materials were composed of HAp, α-TCP, and pectin solutions as a liquid phase. The presence of pectin significantly improved surgical practicality and allowed the production of injectable biomicroconcretes. Studies carried out by immersion in SBF indicated that even after 28 days of incubation, biomicroconcretes maintained their initial shape, indicating high cohesion and resistance to washing. As a result, three phenomena were observed due to the presence of pectin: (i) the increase in the viscosity of the paste, which prevents the penetration of the surrounding medium; (ii) the good interaction between pectin and water, which allows the formation of hydrogels; and (iii) the rapid internal cross-linking of poorly esterified pectins, induced by Ca^2+^ released from α-TCP.^137^

Compression testing showed that the type of pectin (apples or citrus fruits) significantly affects the mechanical properties of the materials. Materials containing citrus fruit pectins showed higher compressive strength values (7.3 MPa). The compressive stress-strain curves of the biomicroconcretes showed that the fracture mechanism of the obtained biomicroconcretes differed compared to the materials. In this sense, materials based on α-TCP showed brittle behavior; however, its presence allowed the materials to be ductile. Furthermore, after the compression test, the biomicroconcretes did not lose their integrity.^137^

The synthesis of pectin-mediated nano-HAp and HAp@pectin nanocomposite by using a carrot pomace pectin as a template was proposed.^138^ Pectin-mediated HAp nanoparticles generated in the presence of an optimal pectin concentration were pure, spherical, low crystalline, and with reduced size. Shape, purity, and crystalline of HAp nanoparticles, and HAp nanoparticles generated in the presence of an optimal pectin concentration and pectin@HAp nanocomposites for bone engineering were improved. Like this, this green pectin-mediated technique produced HAp nanoparticles that can be employed as biomaterials in a variety of biomedical applications.^138^

A sustainable synthesis of nano-HAp using a wet chemical precipitation approach and using biowaste was reported to prepare a HAp compound, obtained from eggshells and banana peel pectin.^139^ The pectin@nano-HAp exhibited characteristic crystallinity and purity with typical hexagonal structure for HAp. FTIR analysis displayed the presence of a calcium-carboxylate group in the pectin@nano-HAp complex. The average hydrodynamic size of pectin@nano-HAp was 208 nm, which was smaller than the hydrodynamic size of HAp (266 nm). The addition of pectin to nano-HAp reduced the size of the particle, and the grain size of pectin@ nano-HAp was 64 nm.^139^

**Figure 12:**
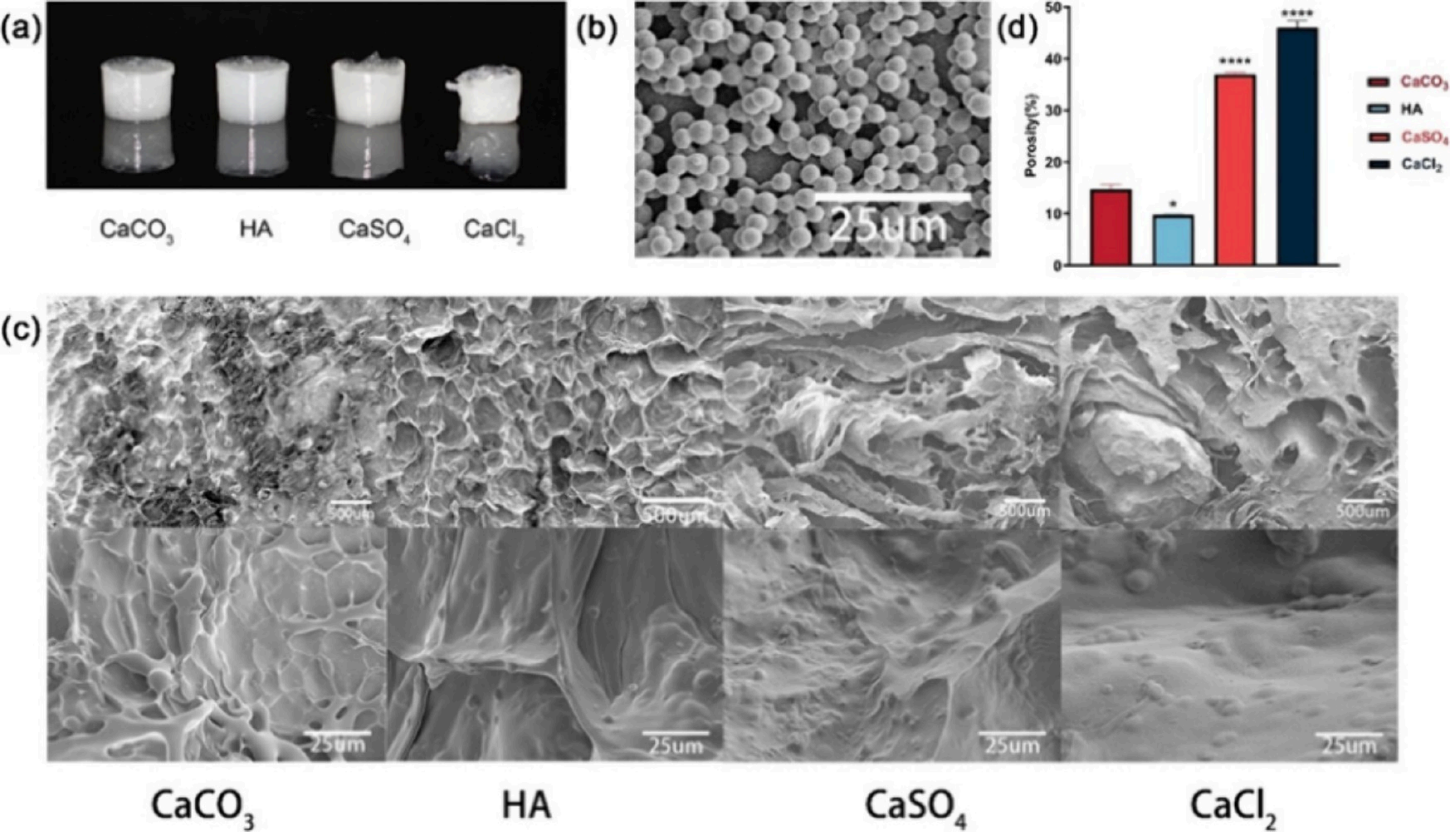
(a) Morphologies of the four hydrogel scaffolds; (b) SEM images of the drug-loaded microspheres; (c) SEM images of the four hydrogel scaffolds with microspheres; (d) porosities of the four hydrogel scaffolds. Reprinted from ref (140). Copyright 2023, with permission from Elsevier.

Different hydrogel scaffolds were obtained of form four different calcium sources (calcium carbonate, HAp, calcium sulfate and calcium chloride) and sodium alginate (Figure 12) were evaluated the differences in their physical properties and the behavior of the scaffolds for bone regeneration and in relation to the in vitro release of the drug naringin, which has antioxidant and anti-inflammatory activities.^140^

Characterizations indicated that HAp-containing materials had the lowest porosity, thus, the highest crosslinking density, which resulted in better mechanical properties and resisted greater compressive stresses (*P* < 0.05). However, the scaffold obtained using CaSO_4_ fractured at 109 kPa, contrasting with the four HAp-containing hydrogels that withstood up to 490 kPa before fracture.^140^ The mechanical properties are related to the cross-linking density of the hydrogel. Furthermore, mechanical properties of the scaffolds when subjected to shear forces through rheological measurements indicated that all four hydrogels were elastic network structures, since a storage modulus (*G*’’) was greater than the loss modulus (*G*′′) for all four scaffolds at frequencies from 1 to 100 rad/s. While the large variation of *G*′ for CaCl_2_ indicated relatively weak stability and in the CaSO_4_ group, *G*’ and *G*′ were within an order of magnitude, indicating poor mechanical properties and consistent with the compression data.^140^

HAp was obtained from fish bone waste and used in the synthesis of composites with alginate and mineral clay montmorillonite (sodium alginate@montmorillonite@HAp) to release doxorubicin and curcumin, a molecule that has medicinal effects to combat various types of cancer, as well as Parkinson’s and Alzheimer’s diseases.^141^

Hydrogels based on alginate, gelatin, and Zn^2+^ doped HAp were prepared for 5-fluorouracil loading, a drug used in chemotherapy. Furthermore, the effect of different concentrations of calcium chloride and glutaraldehyde solutions on hydrogel crosslinking, scaffolds, was also studied and evaluated for drug release.^54^

A stable drug carrier in pH in the gastric environment was proposed.^142^ The composites were based on HAp grafted chitosan@laponite, which exhibits responses depending on the pH of the medium and laponite (a nano clay with high drug transport capacity). The addition of HAp allowed control of the release of the drug in contact with the medium. Structural analysis and swelling tests of the materials indicated that chemical precipitation favored the penetration of HAp nanoparticles into the chitosan and laponite matrix, filling the empty pores. Filling empty pores can lead to the trapping of drug molecules, thus favoring the rate of drug release.^142^

Biocompatible scaffolds were obtained for orthopedic applications based on chitosan@ HAp, in which the chitosan was encapsulated with Mg^2+^, Sr^2+^ doped Hap.^104^ The roughness of the material increased at a higher Mg^2+^ concentration. Furthermore, the presence of Mg^2+^ provided a high resistance to fracture and compressive strength increased from 7.17 ± 1.1 to 15.1 ± 1.5 MPa, and the corrosion behavior of the scaffolds through simulated body fluids showed that the potential corrosion rate was shifted to positive values of 0.14 to 0.002 V after varying the contents of Mg^2+^. Samples with higher crystallographic substitutions showed greater amounts of calcium and phosphate that could be released into the medium, which means a higher dissolution rate than samples with higher crystallinity.

Several chitosan-based composite scaffolds and different concentrations of ceramics were obtained, such as bioactive mesoporous glass and mesoporous HAp, which were synthesized through the freeze-dried process.^143^ The scaffolds have a highly porous structure with interconnected pores and an increase in compressive modulus with increasing ceramic concentration in the scaffolds. The swelling capacity increased in scaffolds without the presence of HAp, ranging between 700 and 900%, and decreased to 400% when the concentration of HAp was higher.

A chitosan-xanthan membrane associated with HAp and different concentrations of graphene oxide was proposed.^144^ The contact angle test indicated the hydrophilic characteristic of all membranes (*p* > 0.05). Polysaccharide-containing materials were more resistant than other membranes.

Polysaccharides from the bone extracellular matrix guide the growth of HAp and allowed several ionic substitutions in Hap.^24^ In this sense, pectin was used in a synthesis to promote the crystallization of Sr-substituted HAp. The preferential affinity of pectin for Sr^2+^ in relation to Ca^2+^ favored the incorporation of Sr^2+^ into apatite, which favored a decrease in the crystal size of the HAp (18.85–26.22 nm) and retained more pectin residues (8–16%). Residual pectin strongly interacted with small HAp particles, resulting in high microhardness (0.43 to 0.85 GPa) and high surface charge (32.1 to 30.3 mV).

Other systems are summarized in Table 3, in which the obtaining methods, general characteristics and evaluated applications are briefly described.

**Table 3:** Methods of Preparation of the HAp Systems, Their General Characteristics, and Applications

System	Method	Characteristics	Application	Reference
HAp@alginate@polyvinylpyrrolidone beads	Precipitation and ionotropic-gelation method	The average particle size of nano-HAp powders was 19.04 nm, and the average bead sizes were 0.98 to 1.23 mm.	Entrapment efficiency and prolonged release of diclofenac	(145)
κ-Carrageenan-crosslinked chitosan@ HAp	Ionically κ-carrageenan-crosslinked chitosan@ HAp hydrogel nanocomposites, by using *in situ* co-precipitation of Ca^2+^ and H_2_PO^4–^ ions	Crystalline hydrogel nanocomposites, with a spherical nano-HAp with size of approximately 20–30 nm and when loaded with ciprofloxacin exhibited activity against Gram-positive *Staphylococcus aureus*	Evaluation of the effect of content of sustained drug release by HAp@ciprofloxacin and antibacterial activity of ciprofloxacin-loaded nanocomposites	(10)
HAp@sodium alginate@ chitosan microspheres	Emulsion method with Ca^2+^ as a cross-linking agent	Good biocompatibility	Doxorubicin hydrochloride delivery and bone tissue engineering	(146)
Alginate@carboxymethyl chitosan@HAp hydrogels for formation of injectable scaffold	Gelling and dual-crosslinked: ionic cross-linking and electrostatic interaction, and also *in situ* synthesized calcium phosphate particles	Particles in the composited gel matrix without agglomeration. The rheological properties, swelling capacity, and degradation were improved.	Pharmaceutical and biomedical drug release (tetracycline hydrochloride and silver sulfadiazine) and as a cell scaffold through behavior in human adipose-derived stem cells of human	(121)
Chitosan@alginate @Fluorine-Hap bioscaffolds	Use of casein micelle-assisted synthesis	The formation of micelles by the casein around the casein-2% Fluorine-HAp and casein-5% Fluorine-HAP crystallites reduced size particles.	Release of ciprofloxacin; future applications in orthopedic implants	(148)
HAp@chitosan three-dimensional composites	*In situ* precipitation of HAp in the presence of chitosan solution, through the solid-liquid method coupled with the freeze-drying process	Compressive strength of the composites was significantly affected by the addition of polymer and antibiotic ciprofloxacin addition. The values obtained are very close to those of cancellous bone tissue (1.5–45 MPa).	Adsorption and release of ciprofloxacin and antibacterial properties	(111)
Chitosan@polylactic acid@nano-HAp scaffolds	3D printing technology	The microstructure of the high porosity composite scaffold with interconnected three-dimensional networks and mechanical properties were enhanced by the addition of chitosan and vancomycin hydrogel.	Dually controlled drug delivery system to vancomycin antibiotic release with the potential to application in infection prevention and large segmental defect reparation	(125)
Alginate@HAp@collagen scaffold	Three-dimensional (3D) printing and freeze-drying process	Scaffold with microporous structures and drug-loaded embedded microspheres into hydrogels with a diameter of 18.62 ± 2.77 μm	Delivery of amoxicillin for repairing infected bone defects	(150)
Nano-HAp@alginate particles	Addition of commercial HAp in alginate solution and by freeze-drying the composite hydrogel	Particles of uniform particle size absorb water rapidly, have easy preservation, higher solubility, and an osteogenic microenvironment.	Evaluation of the biocompatibility and of the hemostasis capacity and the alveolar bone regeneration in tooth extraction wounds	(149)
HAp and β TCP scaffold with a pectin-chitosan PEI coating	HA and β-TCP powders (45 wt %) with a binding agent (poly(vinyl alcohol), 8 wt %), and ultrapure water (47 wt %) to obtain a ceramic slurry. Dolapix CE 64 was added as a dispersing agent Polyurethane sponge impregnation method was used to obtain a macroporous ceramic scaffold. The ceramic-coated sponge was sintered at 1150 °C.	Excellent behavior in a physiological and acidic environment (<10% of mass loss), anti-inflammatory response, and good cell proliferation and migration	Antibacterial material and stimulator of bone formation	(147)

#### 2.3.2 Applications

##### 2.3.2.1 Drug Adsorption and Drug Carrier

A wide variety of composite systems formed based on HAp and polysaccharides have their uses in the adsorption and/or release of different types of drugs such as antibiotics,^150^ anti-inflammatory,^151^ chemotherapeutic and anti-cancer drugs,^55^ among others.

In the last decade, alginate@HAp composites were developed in more complex ternary systems involving other solid matrices,^152,153^ including protein, biopolymers, and oxides. For example, the study by Li et al.^154^ evaluated the HAp effect of the concentration of HAp in alginate-containing composite microspheres for the carriage and release of vancomycin, an important drug with wide applications in the treatment of hard tissue inflammation. Alginate@HAp composites were obtained with a well-defined pore structure of alginate@Sr-HAp, which would normally accelerate drug release from the microspheres, and the composite showed a lower vancomycin release rate.^154^ The formation of a Sr-HAp complex occurred because the presence of Sr-HAp in the spheres restricted the dissolution of the outer alginate shell, and, simultaneously, the alginate in the microspheres protected the interaction between vancomycin and Sr-HAp, leading to a slow rate of drug release. Therefore, the alginate@Sr-HAp composite has better bioactivity and controllable properties for vancomycin release than alginate and Sr-HAp, increasing its potential applications as drug carriers.^154^

Alginate@carboxymethyl chitosan@HAp hydrogels were applied to release tetracycline hydrochloride and silver sulfadiazine, a substance with bacteriostatic action.^121^ A 52.6% release for tetracycline hydrochloride was observed in the first 6 h, which remained between 67 and 69% after one week. Silver sulfadiazine release was 49.2% in the first 24 h, reaching 65% after one week. According to the authors, the releases were 46% for tetracycline and 30% for silver sulfadiazine after the first 24 h, and these results were better compared to hydrogels that contained only alginate.^121^

Scaffolds based on HAp and fluoride-substituted HAp were developed by the sol-gel approach for orthopedic applications. The casein micelle-assisted synthesis route was adopted for the formation of a composite to form HAp, fluoride-HAP, and casein-assisted fluoride-substituted nanoHAp. Composites were applied to load ciprofloxacin, and the antimicrobial efficacy of the scaffolds was also evaluated. Chitosan@alginate@ fluoride-HAp scaffolds showed a ciprofloxacin release of 87.68% in 80 min, while free biopolymer material presented a higher release (92.08% - sintered pure HAp sample) during the first 70 min, with the initial burst of release accounting for 49%. According to the authors, the release of the differences observed in ciprofloxacin can be attributed to the formation of crystalline particles of different sizes as a result of the use of the chitosan and alginate. Furthermore, the formation of micelles by the casein portions around the crystallites formed around the polysaccharides reduced the particle size that influenced the control of the drug release. The biomaterials also showed water absorption, retention capacity, and biodegradability. Antibacterial activity was conducted against *Staphylococcus aureus* (47 mm inhibition zone) and *Escherichia coli* (38 mm inhibition zone), and antifungal activity against *Candida albicans* (10 mm inhibition zone), and the biological efficacy of the casein-5% fluorine-HAp composite was confirmed.^148^

The release of antibiotic amoxicillin by sodium alginate@collagen@HAp microspheres was in the first 96 h, with a complete decrease in 288 h at 0.1430 and 0.2592 mg mL^–1^ drug. Furthermore, the rate and total amount of release gradually increased with increasing drug concentration.^150^

Hydrogel scaffolds formed by sodium alginate combined with four different calcium sources (calcium carbonate, HAp, calcium sulfate, and calcium chloride) were investigated in relation to the in vitro release of the drug naringin. HAp showed a release rate of 15.64% in 24 h. After seven days, the release of naringin reached a plateau of 84.5% for the scaffolds. After 14 days, a small amount of naringin remained on the hydrogel scaffold. Therefore, materials containing calcium carbonate and HAp formed homogeneous hydrogels with better physical and mechanical properties without a change in chemical components. Materials obtained with HAp and calcium sulfate had better controlled release capacity.^140^

The release of diclofenac loaded onto alginate@halloysite@Hap and alginate@halloysite nanotubes (HNT) was evaluated at pH 7.4, 5.0, and 2.1.^155^ A linear release of the drug was observed spheres; the most significant release occurred in the first two hours at pH 2.1, since this is the time required for the granules to pass from the stomach (pH 2.1) to the intestine (pH 7.4). The release of diclofenac of the spheres of alginate at pH 7.4 was 80% in the first 3 h.^155^ After the introduction of HNTs in the spheres, the release rate was 40%, and the release of diclofenac from HNT-containing spheres involved a first desorption of the surfaces of the drug from the external nanotubes and then a second, more prolonged phase, which was dominated by diffusion from the pores inside of the cylinders. The presence of HAp nanoparticles, which were generated *in situ*, restricted the swelling of the granules and the penetration of PO_4_^3–^ into the granules, as well as hindered the dissolution of alginate and diclofenac at pH 7.4.^155^

The adsorption and release of diclofenac were evaluated in the alginate@ polyvinylpyrrolidone@HAp, in which the adsorption of the drug in the composites was better than the materials containing only HAp.^145^ However, when the amounts of polysaccharide and polymer in the composites were lower, the amount of drug carried was higher. In this way, HAp improved the adsorption of the drug; at the same time, a higher amount of HAp favored drug transport into the spheres and decreased its release.^145^

In vitro evaluation at 37 °C of release of aspirin in hydrogel structures based on polyvinyl alcohol, gelatin, sodium alginate, and nano-HAp^151^ showed a cumulative release efficiency between 82.75% and 85.13%. Rapid release occurred within the first 48 h in all materials obtained, which, according to the authors, resulted from the exchange of water molecules in addition to the breakdown of organic chains present in the hydrogel. After 48 h, the release rate was gradually reduced and maintained until 312 h, suggesting that the hydrogel structure maintained a controlled release of aspirin. The results indicated that the hydrogel structure was able to retain drug molecules without excessive loss of aspirin during the preparation process. The presence of nano-HAp increased the efficiency in the participation of drug molecules, ensuring a stable chemical structure, but also favoring drug release kinetics.

The release rates of doxorubicin indicated that drug-loaded alginate@HAp microspheres were effective for a controlled release, which could be attributed not only to the electrostatic interaction between the hydroxyl groups present in the biopolymer but also to the homogeneous dispersion of HAp nanoparticles within the hybrid microspheres and on its surface.^146^

The loading and release of doxorubicin (90.78%) by sodium alginate@montmorillonite@HAp were higher compared to curcumin, with a release also evaluated at pH 5.5, which was (81.18%), pH 6.8 (58.06%), and pH 7.4 (35.08%). The greater release at the evaluated pHs was related to the formation of hydrogen bonds between the OH– group of doxorubicin and the OH– group of the composite, considering that the free −COOH and OH– present in the polysaccharide are protonated at pH 5.5.^141^ Therefore, the functional groups in the polysaccharide present in the sodium alginate@ montmorillonite@HAp composites increased the loading efficiency through a stronger interaction with the clay mineral and the alginate. The cumulative drug release rate decreased when compared with the drug filler content; this may be related to the ionic strength and the presence of the cross-linking agent used in the synthesis. The variation in drug release rates and quantities may be due to swelling behavior and interaction within the drug-polysaccharide matrix. The sodium alginate@montmorillonite@HAp was also applied for the adsorption of curcumin, which was pH dependent. Curcumin releases were 70.12%, 50.85%, and 34.91%. at pH 5.5, 6.8, and 7.4, respectively. The changes in curcumin release at the different pHs were related to the functional group present in the drug molecule. According to the authors, cancer producing cells replicate at pH 5.5, which is the same pH range where curcumin showed better release.^141^

The effect of different concentrations of calcium chloride and glutaraldehyde solutions on the crosslinking of the hydrogel based on alginate, gelatin, and Zn^2+^-doped HAp was evaluated. In addition scaffold preparation and evaluation in drug release were also carried out.^55^ In hydrogel samples prepared cross-linked with formulation I (calcium chloride solution), drug release varied between 56.52 and 66.00% in the first 15 minutes. For samples that were cross-linked with formulation II (calcium chloride and glutaraldehyde solution), the samples showed a release between 50, 34, and 64.55% in the first 15 minutes. All scaffolds showed a rapid release of 5-fluorouracil during the first 10–15 minutes. According to the authors, this rapid release was attributed to the ionotropy between the calcium ions of the alginate and the sodium ions of the phosphate buffer. Phosphate ions caused the dissolution of the alginate structure and, consequently, the release of calcium ions. All scaffolds obtained showed a 100% release of 5-fluorouracil in 4 h. Therefore, drug release obtained in different formulations could be adapted according to the desired use, as a prolonged release of the drug in therapies that require long-term.^55^

Drug delivery systems based on HAp@sodium alginate@Fe_2_O_3_ nanoparticles were prepared by co-precipitation.^157^ The drug loading capacity (catechin hydrate - CH) was 20.31 ± 0.64%, with the maximum drug release obtained at pH 5.5. Iron(III) oxide showed no significant cytotoxic effects. Furthermore, drug-loaded coated nanoparticles showed higher toxicity against HT-29 and MCF-7 cancer cells compared to free cathechin. This *in vitro* study showed that the encapsulation of catechin, as a potent herbal drug, into nanopartical compounds improved its bioavailability, suggesting NPs as an efficient vehicle for targeted drug delivery in cancer treatment.

Chitosan@HAp was used as a vehicle for gentamicin transport and release systems. Release tests showed that there was a controlled release of 42.5 mg of the drug (84.9%) after 24 h.^124^ In a second study, commercial HAp and chitosan were used to prepare scaffolds by different methodologies and apply for vancomycin adsorption and release. The results indicated that 52.5% of the drug was released in 4 h and complete after 24 h.^126^

The release of naproxen sodium from the chitosan@HAp scaffolds^122^ occurred in two different stages: an initial release that refers to rupture during the first 24 h, attributed to the rapid desorption of the drug from the surface of the scaffolds, and a subsequent relatively constant release with a decreasing release rate of the drug that can be attributed to the composition and morphology of the scaffolds.^122^ For example, hydrogen bonds between HAp, chitosan, and the drug in samples containing different amounts of phosphate act as barriers against the release of the drug trapped in the prepared scaffolds. In fact, HAp influenced drug release, and, consequently, a longer period of time was required. Furthermore, the pore size decreased with increasing amounts of HAp in the samples, which may cause a reduction in drug release.^122^

Chitosan@HAp composite containing carrageenan, a sulfated linear polysaccharide used as a cross-linking agent, was applied for the adsorption and release of ciprofloxacin.^10^ In nanocomposites containing HAp, 66% ciprofloxacin was released within 120 h. This phenomenon was attributed to the formation of a compact structure in the nanocomposites, which increases the intermolecular hydrogen interactions between the HAp nanoparticles and the biopolymers, and therefore, ciprofloxacin was strongly maintained in the nanocomposite network.

A chitosan@laponite@HAp nanocomposite showed a high loading capacity for floxacin as a model drug.^142^ The effects of the amount of HAp on the release behavior of the nanocomposite were also investigated in simulated gastric fluids (pH 1.2) and intestinal fluids (pH 7.4).^142^ The ability of a given material to encapsulate a drug is an essential factor in drug delivery systems. In this way, both the unmodified and modified samples presented a high loading capacity (>90%). However, in samples containing only laponite, the drug carrying capacity was higher compared to samples containing chitosan and/or HAp. In laponite, the drug can readily intercalate between layers by ion exchange, which favors a high degree of transport. However, with the addition of chitosan@HAp nanocomposite, a small decrease in loading capacity was observed, which was related to the swelling of the samples at pH 7.4.^142^

A significant decrease in the water adsorption capacity was observed because of addition of the HAp in the chitosan and laponite composites. Swelling rates ranged from 15.1 to 2.5 depending on the amount of HAp. This severe reduction in the hydrophilicity of the products can be attributed to the decrease in pore size caused by the addition of HAp. During the synthesis for the precipitation of HAp nanoparticles, the pH of the solution was adjusted to 10, and ofloxacin is anionic once its p*K*a_2_ is 8.1. Therefore, because the silicate layers are also anionic, repulsion between the anionic species of ofloxacin is dominant. The release was 62.6 and 98.7% for the chitosan and laponite compounds at neutral and pH 1.2, respectively, after 24 h. For nanocomposites containing HAp, the releases were around 18 and 48% at pHs 7.4 and 1.2, respectively. The release rates were slow after 24 h up to 40 days, and no significant changes were observed. This fact was associated with the addition of HAp that reduced the size of the pores in the nanocomposite, trapping the drug molecules, which led to decreases in the rate of diffusion and release of the drug. Electrostatic interactions of phosphate ions with chitosan and in situ precipitation by the addition of Ca^2+^ ions allowed the formation of HAp nanoparticles within the chitosan@laponite matrix, causing the pore size to be reduced.^142^

Studies of the kinetics of adsorption of drug in the two different types of green, sustained, and multifaceted microparticulate hydrogel were created using chitosan, alginate, and doped HAp with the drugs.^127^ A microparticle hydrogel of calcium alginate and calcium alginate chitosan with adsorbed propranolol hydrochloride and sodium salt monohydrate followed a similar behavior, reaching adsorption equilibrium around 2.000 min.^127^ The promising properties of hydrogel microparticles make them a more than adequate candidate for the construction of diverse granular hydrogels with numerous tissue engineering applications, promoting bone healing while avoiding bacterial infection.^127^

When a drug or an active ingredient is introduced into the internal matrix of a polysaccharide, the drug release is governed by water transport. Therefore, hydrogels have distinct and characteristic swelling kinetics, so-called release by swelling-controlled mechanisms, which is mediated by the balance between the diffusion of the drug through the polysaccharide matrix and the opposing flow of water or biological fluid into the polysaccharide. Therefore, the release steps in a hydrogel can be considered as follows: (i) the drug is dispersed in the gel, (ii) the solvent begins to evaporate at the same time that the dissolution medium penetrates the polysaccharide, and (iii) the solvent-free polysaccharide begins to swell. Therefore, depending on the mobility relationship between the dissolution medium and the drug and the swelling dynamics of the polysaccharide, Fickian and non-Fickian transport profiles or anomalous transport profiles may arise.^127,158^

The release rate of adsorbed ciprofloxacin in a three-dimensional chitosan@HAp composite was evaluated for 10 days by in vitro dissolution tests and ranged from 52% to 87%. Antibiotic release profiles as a function of release time are similar and exhibit controlled release. These results can be explained by the modification of the surface morphology of the chitosan@HAp composite.^111^ Furthermore, hydrogen bonding between the antibiotic molecules and the composite can also affect the release kinetics. The release profiles of both formulations showed rapid initial drug release during the first day, about 23%–32%. These high initial release rates could be explained by the dissolution of ciprofloxacin molecules located on the surface of the composites. The drug release profile in PBS buffer revealed a rapid release of approximately 40% in the first 24 h, explained by the weak interactions between the antibiotic and the microspheres. The remaining amount of the drug was released on a sustained profile for 10 days.^111^

After an initial rapid release in the first 3 days of antibiotic vancomycin in the chitosan@ polylactic acid@HAp scaffolds,^125^ a constant release rate was followed. Furthermore, the scaffolds produced controlled release *in vitro* for more than 8 weeks. Therefore, on day 15, the amount of controlled release in the scaffolds varied between 64.7 and 90%. On the 30th day, the values varied between 73.8 and 93.5%. Therefore, the hybrid scaffolds presented better controlled release capacities compared to hydrogels containing only chitosan and vancomycin.^125^

Chitosan@silylated HAp cross-linked with glurathaldehyde nanocomposites adsorbed large amounts (125 mg g^–1^) of diclofenac in 15 min. The formation of chitosan@HAp nanocomposites involved interaction between the amine groups present in chitosan, amino HAp, and the aldehyde groups present in the bifunctional agent.^159^

The amount of the analgesic tramadol released gradually increased in mesoporous chitosan@SiO_2_@HAp and reached between 30 and 58% after 24 h. According to the standard system implanted in the damaged area, approximately 280 μg of tramadol can be released per minute. Therefore, the release is within the therapeutic range proposed for the drug. According to the authors, using chitosan@SiO_2_@HAp structures loaded with the analgesic is a good system for tramadol emission, as the amount of drug released from the scaffolds reached 98% after 240 h, with limited side effects.^160^

Antimicrobial properties of the K2 vitamin, nano-HAp, and chitosan-coated dental implants nanocomposite against clinically relevant microbial strains against *Staphylococcus aureus*, *Streptococcus mutans*, *Enterococcus faecalis*, and *Candida albicans* were studied by using the agar well diffusion test. The K2@nHAp nanocomposite exhibited antimicrobial activity against all microorganisms, showing the highest sensitivity against *E. faecalis* (inhibition zone of 25 mm at 100 μL). However, the K2@chitosan@nano-HAp nanocomposite demonstrated potent antimicrobial activity against *C. albicans* exhibiting the highest sensitivity (28 mm inhibition zone at 100 μL concentration). Vitamin K2 showed limited antimicrobial activity, and vitamin K2@chitosan exhibited significant susceptibility to *C. albicans*, resulting in a substantial inhibition zone of 24 mm in diameter at a concentration of 100 μL. Therefore, the synergistic effects of vitamin K2, nano-HAp, and chitosan highlighted the potential of the obtained nanocomposites for biomedical applications.^161^

Cellulose nanocrystals are expected to be used for biomedical applications, especially for hard tissue engineering, because of their low toxicity to the human body and exceptional physicochemical properties. In this sense, cellulose nanocrystals@HAp nanocomposites resulted in a high mechanical resistance and biocompatibility for their use as a biocompatible dental restorative material. However, the high hydrophilicity of the cellulose nanocrystals@HAp has limited the restorative function of HAp. As an alternative, a chitosan@cellulose nanocrystals@HAp was prepared and applied as new dental biomaterials. The preparation involved the mixing of cellulose nanocrystals@HAp organic-inorganic particles with a chitosan matrix. In this way, the hydrophobicity of the chitosan@cellulose nanocrystals@HAp nanocomposite was drastically improved, which consequently protected the sample from deterioration in water. Immersion of chitosan@cellulose nanocrystals@HAp in artificial saliva confirmed that the HAp layer was formed by remineralization after immersion. Furthermore, the amount of HAp increased with increasing immersion time in artificial saliva evaluated by thermogravimetry. Therefore, chitosan@cellulose nanocrystals@HAp promoted remineralization and can be applied as a dental material with self-healing properties due to its ability to mediate remineralization.^162^

Osteochondral grafts are used to repair focal osteochondral lesions. Autologous grafts are the standard treatment; however, the limited availability of grafts and donor site morbidity restrict their use. Therefore, there is a clinical demand for different sources/materials of grafts that reproduce the natural function of cartilage, and chitosan has been proposed as an alternative in this field. In this sense, the biomechanics and biotribology of a bioabsorbable chitosan@nano-Hap osteochondral construct were implanted in an experimental model for *in vitro* porcine knee simulation. The osteochondral construct implanted in different surgical positions (level, proud, and inverted) was compared with grafts in current clinical use and a positive control consisting of a stainless-steel graft implanted on the cartilage surface. After 3 h (10,800 cycles) of wear simulation under walking, subsidence occurred in all osteochondral construct samples, regardless of surgical positioning, but without apparent loss of material and low wear of the meniscus. Half of the predicated grafts exhibited delamination and scratches on the cartilage surfaces. No graft subsidence occurred in positive controls, but meniscus wear and deformation were evident. Implantation of a new chitosan-based osteochondral construct ideally shaped (flush), inverted, or proud of the cartilage surface resulted in minimal wear, damage, and deformation of the meniscus.^163^

A nanocarrier was developed through the emulsification method using chitosan, HAp, and graphitic carbon nitride (g-C_3_N_4_) and was used for transport efficiency and controlled release of 5-fluorouracil. Synthesis involved incorporation of the g-C_3_N_4_ nanosheets into the chitosan@HAp hydrogel. The drug loading efficiency and entrapment efficiency reached 48% and 87%, respectively, and FTIR and XRD indicated the chemical interaction between the drug and the nanocarrier. The biocompatibility of the g-C_3_N_4_@chitosan@HAp nanocomposite against MCF-7 cells was demonstrated by the MTT method and confirmed by flow cytometry. In this sense, the nanocomposite carried with 5-fluorouracil led to a higher rate of apoptosis in MCF-7 cells, indicating the efficiency of the nanocarrier in killing cancer cells, demonstrating potential for the treatment of cancer cells.^164^

Although pectin has properties that enable the entrapment and/or delivery of drugs, few studies were found focused on pectine@HAp nancomposites for absorption and release of pharmaceuticals to be addressed within the scope of this review.

The slow gelation kinetics of hydrogels is necessary for the loading of cells, drugs, or other bioactive molecules because of the technical time required for the immobilization process. Evaluation of the *in vitro* stability/dissolution kinetics of pectin hydrogels can be useful to predict the *in vivo* degradation behavior of biomaterials and their dissolutions.^128^ The reversibility of the dissolution of pectin@HAp hydrogels was obtained with 0.9% NaCl solutions, possibly to accelerate the release of cells or immobilized active ingredients. The results obtained support the hypothesis that the gel network can be easily dissolved due to the competitive binding between sodium and calcium ions and the consequent disruption of the egg-box structure of the gel. This result is particularly interesting for externally modulating the release of drugs or possibly immobilized cells or for dissolving and removing the gel in the event of adverse reactions.^128^ However, typical solutions used *in vitro* as the incubation medium (culture medium, aqueous solutions, and saline solutions) are far from resembling physiological fluids. Therefore, more studies are needed on the *in vivo* degradation behavior of pectin@HAp hydrogels.^128^

The amount of cellulose nanocrystals in the hydrogels with HAp improved the thermal stability, mechanical properties, and mineralization of HAp by the biomimetic method, where simulated body fluid was used. Hydrogels with 5% cellulose nanocrystals showed a higher amount of HAp, where they were immersed for 14 days with 28% HAp. Compared to hydrogels in which HAp was added by chemical precipitation, they contained 20% of the phosphate nanoparticles. The biocompatibility of the hydrogels was assessed by cell viability using fibroblasts (L929). In general, hydrogels obtained by the biomimetic method showed slightly higher biocompatibility compared to hybrid hydrogels obtained by chemical precipitation.^129^

In the last decade, a single study was found that reported the acquisition of scaffolds containing the combination HAp and β-TCP forming composite systems with pectin and chitosan for drug transport. Initially, for scaffolds containing calcium phosphates and vancomycin, an intense release was recorded between 4 and 8 h, which was due to the high solubility of vancomycin, since no type of interaction or encapsulation occurred, but simply by adsorption. On the surface of the ceramics, therefore, after immersion in physiological solution, all antibiotics are released in a few hours. The addition of pectin to prepare the scaffolds allowed for a prolonged retention of vancomycin; however, under physiological conditions, pectin strongly adsorbs water. Therefore, the solution infiltrated into the scaffolds caused an intense release in the first 24 h. However, with the addition of chitosan, the structures presented a controlled and prolonged release of up to 2 weeks, and the formation of the polyelectrolyte complex resulted in a designed coating that controls vancomycin release. The presence of the polyelectrolyte network allowed vancomycin encapsulation and slow degradation of the coating, which allowed for prolonged release to be achieved, which is favorable for controlling bacterial infection and preventing periprosthetic infections.^147^

Cathodic electrophoretic deposition was used to fabricate novel chitosan@pectin@HAp porous nanocomposite coatings on Ti6Al4V substrates. The Rietveld refinement indicated variations in structural changes resulting from reducing the applied voltage from 30 to 10 volts. Deposits obtained at 10 volts after 1 h of pre-sedimentation exhibited a dense microstructure with a narrow pore size distribution compared to others. The wettability and adhesion strength of coated Ti6Al4V improved with increasing HAp content. The release of the vancomycin from the composite coating confirmed a 40% initial release, 50% semistable release, and 10% residual release. A thick and uniform layer of porous apatite was formed in Ti6Al4V after 21 days of immersion in simulated body fluid.^156^

##### 2.3.2.2 Bone Tissue Engineering

A very promising method of filling bone defects caused by fractures, congenital osteogenesis imperfecta, bone neoplasms, and trauma is the use of bone grafts to support orthopedic or dental implants, allowing a significant improvement in patient quality of life.^112^

Alginate@HAp hydrogels were obtained for application in bone regeneration.^116,165−167^ Membranes for guided bone regeneration should have a mechanical structure and a chemical composition suitable for mimicking biological structures.^130^ A periosteum-inspired bilayered membranes obtained by crosslinking alginate with different amounts of nano-HAp. The ionic interaction between alginate and nano-HAp influenced the strength and microstructure of the hydrogels. Distinct surface characteristics were achieved on each side of the membranes, resulting in a highly porous fibrous side and a mineral-rich side with higher roughness and lower porosity. Moreover, the effect of amount of nano-Hap decreased the membranes’ plasticity and an increment of degradation rate. The authors evaluated cells similar to osteoblasts that proliferated and differentiated on the mineral-rich side, especially in higher amounts of nano-HAp, while cells similar to fibroblasts were able to proliferate on the fibrous side, which favors the use of these membranes as systems of bone repair.^130^

The swelling rate of different hydrogel scaffolds obtained with four sources of calcium (calcium carbonate, HAp, calcium sulfate, and calcium chloride) and sodium alginate^140^ is directly reflected in the efficiency of material metabolism, but excess swelling affects bone tissue growth and reconstruction. In this sense, the degradation of the hydrogel scaffold was very important in bone regeneration. However, the mass of each group decreased with time, as the degradation rates of the four scaffolds reached ∼50% after 15 days. Due to the exchange of calcium ions with other ions, the degradation rate depended on the exchange rate of calcium ions.^140^

Dental extractions can lead to complications such as post-extraction bleeding and bone resorption, and also to unfavorable results for subsequent implant restoration. Therefore, additional restorative procedures, such as hemostasis or bone regeneration, were proposed that evaluated the biocompatibility and hemostatic capacity of HAp and alginate particles in a rat tooth extraction model.^149^ The results indicated cell viability greater than 90% in all experimental concentrations evaluated, in which the absence of red coloration in cellular fluorescence images indicated viability in the majority of cells, in addition to the cytocompatibility of the materials obtained. Excellent *in vitro* hemocompatibility of the particles was observed, since hemolytic activity assays resulted in hemolysis rates below 2.0% in all samples.^149^

According to authors,^149^ tissue engineering materials play a critical role in the regeneration and reconstruction of bone defects. The ideal material for bone tissue engineering should not only be biocompatible but also promote the differentiation of precursor cells into osteoblasts to create a favorable osteogenic microenvironment. Therefore, to evaluate the osteogenic induction capacity of the materials, the authors performed in vitro alkaline phosphatases (ALP) and alizarin red S (ARS) staining tests. ALP is the first marker of osteogenic differentiation, while ARS staining is a late marker of mineralization. As a result, the alginate@HAp nanocomposite showed the highest staining intensity with the highest proportion of positively stained areas, indicating greater osteogenic differentiation of precursor cells. It indicates that the nanocomposite can effectively promote the differentiation of bone marrow mesenchymal stem cells into osteoblasts, thus improving the mineralization of bone tissue.^149^

The effect of filling surgical sites with alginate@HAp nanocomposite after extraction was evaluated after 28 days. In the group where the nanocomposite was used, a greater proportion of new bone was formed around the alveolar fossa, filling the extraction sockets uniformly, in addition to showing superior osteogenesis in the external area of the socket. After 28 days, the presence of HAp in the alginate@HAp nanocomposite group promoted bone regeneration, showing significantly higher X-ray opacity in the extraction sockets compared to the other evaluated groups.^149^ The study suggested a promising alternative for clinical applications in hemostasis and bone regeneration after tooth extraction.^149^

Spheres of nanometric size for tissue repair were obtained in an alginate@HAp by using an HAp from poultry and shellfish by-products. The ability of beads to absorb body fluid was evaluated, as it is an important parameter not only for mimicking the original cell environment but also for modulating the mass transfer properties. The absorption capacity of the particles was between 77 and 223% of their mass, a positive indicator of their ability to promote tissue regeneration.^131^

Among the various tests performed with the different silk fibroin@alginate@HAp scaffolds, its effects on bone marrow mesenchymal stem cell proliferation assays are worth highlighting.^132^ The optical density of cultured cells increased positively with the number of cells. After 3 days of culture, the effect on cell proliferation was significantly greater as the amount of silk fibroin increased in the scaffolds. This can still be observed because the culture time was longer. The result of cell life and death after 3 days of culture allowed us to conclude that the compound provided good growth conditions, in addition to a state of propagation.^132^

The cytocompatibility of the alginate@HAp microsphere scaffolds loaded with amoxicillin and collagen was evaluated by seeding rabbit adipose tissue-derived stem cells, and the results proved that the cells could attach, proliferate, and migrate on the scaffold and exhibit favorable cytocompatibility. The results demonstrated that scaffold preparation was feasible and the scaffold has great potential for the repair of infected bone defects.^20^

The versatility of the materials for bone implants is of fundamental importance in order to adapt to the location where it is being implemented. In this sense, scaffolds were produced from a hydrogel composed of a sodium alginate with HAp reinforcements using a 3D bioprinter for the regeneration of bone tissue.^168^ The alginate solution was prepared by dissolving alginate and HAp in mass/volume proportions of 2.5% and 5.0% and also using a calcium chloride solution. Morphological characteristics, physicochemical properties, and biological responses of the scaffolds were analyzed as a function of the HAp concentration. The incorporation of HAp into the alginate matrix and formation of the hydrogel were confirmed. Preliminary analyses indicated that scaffolds containing 2.5% HAp were within the cytotoxicity limit (66.4 ± 7.0%) for cells of the canine E20 lineage. On the contrary, scaffolds with 0% and 5.0% HAp were not cytotoxic. In fact, the latter structure demonstrated greater cell proliferation, as predicted, due to the hydrophilic properties of sodium alginate that allow easy and rapid cell seeding, facilitating nutrient transport and cell growth within the structure.^168^

Alginate@graphene oxide@sericin@nano-HAp nanocomposite hydrogels were used to explore the effect of the hydrogel with osteoimmunomodulatory properties on the promotion of osteogenesis of bone marrow stem cells (BMSCs).^169^*In vitro* experiments revealed that the hydrogel presented desirable mechanical strength, stability, porosity, and biocompatibility. Significantly, sericin and nano-HAp appeared to exert synergistic effects on bone regeneration. Sericin was observed to inhibit the immune response by inducing macrophage M2-type polarization to create a positive osteoimmune microenvironment, helping to improving osseointegration at the bone-implant interface to promote osteogenesis. However, osteogenic differentiation in rat BMSCs was further enhanced by combining nano-HAp and sericin in the nanocomposite hydrogel. Eventually, the hydrogel was implanted in the rat cranial defect model, assisting in the reduction of local inflammation and efficient bone regeneration. The nanocomposite hydrogel stimulated bone formation by the synergistic effects of immunomodulation of macrophage polarization by sericin and direct osteogenic induction by nano-HAp, demonstrating that such a scaffold that modulates the osteoimmune microenvironment to promote osteogenesis is a promising approach for the development of bone tissue engineering implants in the future.^169^

Osteoplastic composites with an experimentally determined content (375 μg/g) of the micro (ZnOMPs) and nano (ZnONPs) particles on HAp@alginate@chitosan were obtained.^170^ The ZnONPs showed pronounced antimicrobial activity against *E. coli* (ATCC 25922) and *S. aureus* (ATCC 25923), while ZnOMPs showed activity only in the presence of chitosan. Composites containing ZnONPs/MPs did not have a toxic effect on bone-forming cells, osteoblasts, preserving their ability to biomineralize. ZnOMPs and ZnONPs to varying degrees, but significantly, affect the swelling, porosity, and shape stability, and prolong the release of vitamin D3 for 120 h, compared to control. The biocompatibility and lack of toxic effects give both composites a perspective for osteoplastic application, but ZnONPs@composites were more attractive.^170^

Ions such as Mg^2+^, Sr^2+^, Ca^2+^, and P^5+^ in chitosan@HAp nanocomposites favored cell growth and tissue regeneration, and the content of the ions can be higher depending on the dissolution rate, which is accompanied by low crystallinity and a high content of ionic substitution. Therefore, the manipulation of biocompatibility can be controlled through compositional constituents. Furthermore, the biocomposite based on chitosan and Mg^2+^, Sr^2+^ doped HAp showed better cell survival in cell viability, reaching a value of 105.2 ± 6.8% for materials with the highest amounts of Mg^2+^. Since the concentration showed a statistically significant positive correlation (*r* = 0.989; *p* = 0.0014) with the percentage of cell viability, it reflected greater biocompatibility.^104^

Drug-bioactive-loaded porous scaffolds incorporating nano-HAp, chitosan, and either hydroxypropyl methylcellulose or silk fibroin (*Bombyx mori*) were fabricated through the freeze-drying method as a subchondral bone substitute.^134^ Drug release data on the porous scaffolds of the new chitosan@nano-HAp composite indicated that the composites were loaded with drugs with triamcinolone acetonide or transforming growth factor-β1, respectively. The growth factor was released at a controlled rate rather than burst release, which could be helpful in reducing post-surgical medication administration, and a reduction in the release rate can be achieved through incorporation of the drug and/or growth factor into microspheres. The cytocompatibility of the tissue scaffolds was evaluated by cell adhesion and viability tests, and PCR data showed that the addition of an anti-inflammatory drug can reduce the chances of inflammation at the defect site, and the pro-osteogenic activity can be improved by incorporation of triamcinolone acetonide or transforming growth factor-β1 in the tissue scaffolds. The authors concluded that such biomaterials have long-term potential for clinical applications in the field of subchondral tissue regeneration.^134^

The presence of nano-Hap in the chitosan@nano-HAp scaffolds promoted a significantly lower biodegradation rate compared to that of a scaffold containing only chitosan when evaluated in the simulated fluid.^134^ Both types of scaffold significantly inhibited the growth, fixation, and colony formation of *S. aureus* and *E. coli*, increasing the relevance of chitosan in the composition of the grafts for the naturally contaminated oral environment. Confocal microscopy analysis showed MG-63 cells with normal morphology and indicating adherence and proliferation within the porous structure of the biomaterials, especially for the chitosan@nano-HAp scaffold, which reached a higher proliferative rate in 14 days. MG-63 cells seeded within chitosan@nano-HAp scaffolds showed a higher expression of the osteogenic genes RUNX2, collagen A1 and Sp7 compared to the chitosan samples. *In vivo* subcutaneous implantation in mice of both types of scaffolds showed lower biodegradability with preservation of the porous structure that allowed the growth of connective tissue up to 5 weeks. Histology shows an intensive and progressive growth of new vessels and collagen between the first and fifth week, especially for the chitosan@nano-HAp structure.^134^

A collagen@HAp@chitosan composite was prepared and applied as bone matrix to stimulate ossification.^171^ The use of natural sources of HAp and chitosan derived from sea cucumber and shrimp shells were evaluated and levels of cytokines, polymorphonuclear neutrophils (PMN), serum liver enzymes, calcium, phosphate, and procollagen-like propeptide were quantified. 1 N-terminal (PINP) in albino rats with femoral bone defects were also followed. The presence of chitosan@HAp composite reduced the amount of cytokines during the recovery period. Polymorphonuclear neutrophil levels increased in the initial period and then gradually decreased until 42 days of the healing period. A significant decrease in cytokine and PMN levels occurred between 7 and 42 days due to the presence of the scaffold. However, calcium and phosphate levels and PINP levels increased significantly over the same period. Regarding serum liver enzymes, alkaline phosphatase levels in scaffolds increased significantly at 42 days.^171^

Chitosan composite scaffolds with bioactive glass and HAp behaved as the best combination because of their better performance in bone tissue engineering.^143^ All scaffolds obtained from chitosan-based composites and different concentrations of bioactive mesoporous glass and mesoporous HAp were not cytotoxic at 12.5 mg mL^–1^ and showed better cell adhesion and proliferation.^143^

The hydrophilicity of tissue engineering structures is important for improving cell viability and proliferative capacity, as well as allowing body fluids, proteins, and cells to penetrate the structures, thus promoting the growth of new bone tissue. HAp has good hydrophilicity due to the presence of the hydroxyl group on its surface, and according to the authors, the chitosan@HAp scaffolds loaded with plaque-rich fibrin had a strong water absorption capacity, with rates exceeding 300%. However, with an increase in the load of freeze-dried plaque-rich fibrin, the water absorption capacity of the scaffolds decreased slightly, but showed a statistical difference between the groups (*p* > 0.05).^22^ The release of growth factor loaded with freeze-dried plaque-rich fibrin was significantly prolonged, up to 35 days.^22^

A biogenic HAp@chitosan composite may be a valid future candidate for bone tissue regeneration, especially cortical bone. Bioactivity was demonstrated by SBF immersion for 24 h, revealing the formation of a thin, widespread thin layer of apatite on the surface of the samples.^9^

The scaffolds formed by nano-HAp, gelatin, chitosan, and polyvinyl alcohol effectively promoted cell proliferation and adhesion, which were shown to contain 12.5% nano-HAp and had a high capacity for osteogenic differentiation.^3^

The corrosion potential of the composite coatings increased compared to that of pure 316L stainless steel. HAp crystals formed from the chitosan@gelatin@HAp composite after immersion in SBF, confirmed the bioactive nature of the coating. The coating was cytocompatible and allowed the growth and proliferation of osteoblastic cells.^172^

HAp deposition was observed in all membranes after the bioactivity test, where the cell viability of the chitosan@xanthan@HAp@graphene oxide material was higher than that of materials containing only polysaccharides. However, the addition of HAp and graphene oxide reduced the mechanical resistance of the membranes and improved their cell viability.^144^

The degradation rate and mineralization capacity of the carboxymethyl chitosan@HAp scaffolds increased as the carboxymethyl chitosan content increased, but the compressive strength during degradation also increased. Cytotoxicity, cell adhesion, and cell proliferation tests showed that composite samples containing 3% carboxymethyl chitosan resulted in better bioactivity, indicating that the incorporation of the polysaccharide into phosphate can improve not only the mechanical property but also the biological activity.^136^

The osseointegration of dental implants and their consequent long-term success are guaranteed as long as there is enough healthy alveolar bone. Bone deficiencies can be the result of extraction trauma, periodontal disease, and infection. In these cases, titanium implant placement is indicated when the implant allows vertical bone augmentation. This objective was only achieved when the materials used for the bone graft simulate the extracellular matrix and, in this way, promote osteoblast proliferation, which maintain the space without collapsing until the formation of new bone.^115^ Therefore, a moldable chitosan@pectin hydrogel reinforced with HAp and β-TCP particles with a size in the range of 100–300 μm obtained. The polysaccharide nature of the hydrogel obtained mimicked the extracellular matrix of natural bone, and the ceramic particles promoted high osteoblast proliferation. The swelling properties allowed significant adsorption of the aqueous solution (up to 200% of the solution content) so that the space between bone defects can be filled with the material in an in vivo setting. After 6 h, the swelling rate began to decrease due to initial degradation of the network. As the p*K*_a_ of pectin is 4.0 and that of chitosan is 6.0, at a pH of 5.5, more than 99% of the pectin is still in its ionized form, and chitosan exists both in its ionized form (NH_3_^+^) and in its non-ionized form (NH_2_). Due to intramolecular hydrogen bonds between the -COOH_3_ and OH groups in the network at pH 5.5, the matrix was more stable, and the pectin chitosan hydrogel after 24 h at pH 5.5 showed a significant degree of swelling, compared to the other pH values. Furthermore, cellular studies with the human osteoblast line SAOS-2 show high cell proliferation and adhesion already after 72 h, and the presence of ceramic particles increases the expression of alkaline phosphatase activity after 1 week. These results suggest a good potential for moldable biomaterials developed for alveolar bone regeneration.^115^

The cashew tree gum, *Anarcadium occidentale L.*, is a polysaccharide material highly available in the Northeast region of Brazil and was used enriched with HAp to origin a new scaffold, with the objective of assessing the biocompatibility with human tissues and the possible cytotoxicity in murine adipose-derived stem cells cultures. The scaffold has favorable macro- and microscopic characteristics for potential use as a support matrix and growth of adipose-derived stem cells. It did not show toxic effects *in vitro* and induced an increase in cell viability.^173^

Polysaccharide-based membranes to be used in guided tissue and bone regeneration were prepared using chitosan and xanthan gum in the presence of HAp.^174^ HAp was used as a potential drug carrier and also to improve the bioactivity and biomimetic properties of membranes. The FTIR and XRD results indicated the successful incorporation of HAp into the membranes, without significant changes in the crystal structure after incorporation of the polysaccharides. The membranes produced presented asymmetrical surfaces, with distinct roughness, due to the increased concentration of HAp. Chitosan@xanthan@HAp membranes showed higher proliferation *in vitro* of dental pulp mesenchymal stem cells. The results suggest that the addition of HAp to membranes influenced mechanical parameters, as well as cell adhesion and proliferation, supporting the potential application of these materials in regenerative techniques and in the treatment of periodontal lesions.^174^

Currently, bionatural injectable hydrogels are receiving attention due to their ability to control, adjust, and adapt to random bone defects, as well as their ability to mimic the composition of natural bones. Therefore, an injectable hydrogel paste based on natural alginate (from brown seaweed) reacted with a biogenic nano-Hap (from eggshells) and was enriched with valuable trace elements. The alginate@nano-Hap hydrogel showed good biodegradability and satisfactory bioactivity, allowing the progress of angiogenesis, endochondral ossification, and osteogenesis throughout the defect area, positively impacts healing time, and ensures complete restoration of well-mature bone tissue, similar to natural bone.^175^

In the biomedical field, nano-Hap is still one of the most attractive candidates as a bone substitute material because of its similarity to bone mineral characteristics. Ion substitution and low crystallinity are also fundamental characteristics of bone apatite, making it metastable, bioabsorbable, and reactive. In this sense, biomimetic composites of apatite and apatite@chitosan were produced by dissolution-precipitation synthesis using mussel shells as a biogenic source of calcium. Apatite@chitosan composites were also loaded with strontium ranelate, an antiosteoporotic drug. As a result of the metastability and temperature sensitivity of the produced composites, sintering was performed by conventional methods, and therefore cold sintering was selected for the densification of the materials. Composites were consolidated to ∼90% relative density by applying 1.5 GPa uniaxial pressure at room temperature for 10 min. Both the synthesized powders and the cold sintered samples resulted in biomimetic apatite@chitosan composites. Preliminary in vitro tests indicated sustained release of strontium ranelate for approximately 19 days and no cytotoxicity in human osteoblast-like cells (MG63) exposed for up to 72 h to the compound extract containing the drug.^176^

Repairing defects in the alveolar bone is essential for regeneration of periodontal tissue; however, it is a challenge. A promising therapeutic approach involves the use of a strategy that specifically recruits periodontal ligament cells with high regenerative potential to achieve in situ regeneration of alveolar bone. In this study, a nano-HAp@chitosan microsphere conjugated with an antibody was developed to target the p75 neurotrophin receptor (p75NTR). The goal was to selectively attract p75NTR+periodontal ligament cells and promote osteogenesis. *In vitro* experiments demonstrated that antibody-conjugated microspheres attracted significantly more cells compared to unconjugated microspheres. Incorporation increased the cell adhesion and proliferation of cells on the surface of the microsphere, and it also improved its osteoinductive properties. This chitosan@HAp microsphere conjugated with p75NTR antibody presents a promising approach to selectively recruit cells and repair bone defects.^177^

Nanofibrous mats were obtained by mixing cellulose nancocrystalline and nano-HAp in the presence of chitosan and gelatine solutions by electrospinning. HAp and cellulose nancocrystalline were used as filler materials in the nanofibrous blankets. Furthermore, the polymer chains of gelatin and chitosan were cross-linked with glutaraldehyde. The diameter decreased from 86 to 43 nm with increasing electrical conductivity of the spinning solution from 890 to 1166 μS cm^–1^, and after crosslinking, a significant variation in fiber was observed. The blankets presented single-phase transition temperatures in the DSC analysis, which shows that there was no segregation of materials in the electrospun fibers. Cytotoxicity analysis of the vero-cell lineage mats showed around 95% cell viability. The prepared mats were applied as bandages in a mouse model experiment, and 50% faster wound healing was observed in the mice for the noncross-linked mats than for the control.^178^

Healing significant segmental bone defects remains a challenge, and several studies attempt to produce materials that mimic bone structures and properties compatible with native bone tissues. In this way, a nanofiber based on polyvinyl alcohol, polyvinylpyrrolidone, and chitosan was prepared by the electrospinning method and, in combination with HAp, was synthesized to mimic the extracellular matrix. HAp was obtained from lobster shells (*Panulirus homarus*, SL) as a source of calcium. The use of higher concentrations of HAp decreased the diameter of the fiber and improved the mechanical properties of the nanofiber. Furthermore, water absorption increased as a result of greater hydrophilicity at higher concentrations of HAp, which led to an improvement in the nanofiber protein degradation and adsorption process. Biomineralization in a simulated body fluid (SBF) solution confirmed that HAp in the nanofiber increased bioactivity and HAp formation increased during prolonged immersion in the SBF solution. The HAp nanofiber presented a higher potential for osteoblast cell viability after incubation for 24 h, which allowed cell attachment and proliferation. Furthermore, the higher concentration of HAp in nanofibers can significantly promote osteogenic differentiation of MC3T3E1 cells.^179^

The combination of cells and biomaterials has become a powerful approach to regenerative medicine in recent years, and understanding the *in vitro* interactions between cells and biomaterials is crucial to the success of regenerative medicine. Therefore, a scaffold containing stem cells derived from adipose tissue, polysaccharides (pectin, chitosan, and cellulose), and nano-HAp was applied to carry the bone resorption alendronate. The immunomodulatory properties and biological behaviors of mesenchymal stem cells were evaluated for bone tissue repair. The scaffolds showed improved proliferation and viability of mesenchymal stem cells compared to other conditions and a significant increase in gene expression and protein levels of the anti-inflammatory cytokines TGF-β, HGF and IDO in the presence of the alendronate-loaded scaffold nano-HAP, indicating an increase in immunosuppressive activity of mesenchymal stem cells *in vitro*.^180^

Bone transplantation is the second most common transplant surgery in the world. Therefore, artificial bone transplantation to repair bone defects is an urgent issue. In bone tissue engineering, HAp plays an important role in bone graft applications. In this sense, hexagonal HAp nanorods are obtained from shells in a solid-state hydrothermal transition process. HAp nanorods (∼2.29 nm) were reinforced with carbon nanotubes and chitosan on polymer-supported graphene oxide sheets by an in situ synthetic approach. Among the synthesized nanocomposites, the HAp, oxide graphene, chitosan, carbon nanotubes, and polylactic acid composite presents micro- and macroporosity (∼200 to 600 μm), greater mechanical resistance, (Hardness ∼90.5 MPa; Tensile strength 25.62 MPa), and maximum cell viability in osteoblast-like cells of MG63 (80%).^181^

Scaffolds with various proportions of chitosan and mesoporous HAp were obtained, and mesoporous Hap@SiO_2_ nanocomposites were produced by freeze drying for use in bone tissue engineering. The addition of silica-containing mesoporous HAp particles resulted in a structure with uniformly distributed pores, subsequently leading to reduced rates of biodegradation and water absorption in the scaffolds. The introduction of mesoporous Hap particles notably improved the surface coverage of the scaffold with apatite films. Furthermore, biocompatibility evaluations using the osteogenic sarcoma cell line (SAOS-2) highlighted the positive impact on cell adhesion and growth. Spindle cells with a greater number and normal cell nuclei for scaffolds containing mesoporous SiO_2_@HAp particles were observed by fluorescence images. MTT evaluation indicated that scaffolds containing mesoporous particles had approximately 25% more surviving cells compared to scaffolds containing chitosan@HAp. Mesoporous structures showed better activity in relation to the alkaline phosphatase test. The chitosan@silica@HAp scaffolds did not show signs of early or late apoptosis of SAOS-2 cells.^182^

Chitosan@ZnO@HAp crosslinked with glutaraldehyde was reported as potential systems for scaffolds or surface coating systems on dental and medical implants to improve infection processes and biocompatibility.^183^

Chitosan@ZnO@HAp^184^ and chitosan@ZnO@HAp biocomposite was obtained to evaluate and examine its antimicrobial activity *in vitro* against several fungal phytopathogens, and in planta against *Pseudomonas syringae* pv.

Collagen@chitosan@HAp@Mg@ZnO scaffolds were prepared in different mass proportions.^185^ Subsequently, the scaffolds were subjected to gamma radiation aiming at the physical crosslinking of the polymeric matrix, which improved mechanical strength (0.82 to 1.86 N/mm^2^) increasing the thickness of the pore wall. The irradiated and nonirradiated scaffolds were biocompatible and noncytotoxic to the cell line, which ensured their suitability for in vivo use. These results demonstrated that the sterilization of structures obtained with gamma irradiation substantially improved the physicochemical and morphological characteristics, which favors its use in the regeneration of bone tissue and may support the formation of new bone.

The physical and biological properties of osteoplastic composites synthesized with micro- and nanoparticles of ZnO immobilized on the alginate@chitosan@HAp were evaluated.^170^ The composites obtained did not have a toxic effect on bone-forming cells, osteoblasts, preserving their biomineralization capacity. The amount of micro- and nano-ZnO particles significantly affected swelling, porosity, and increased Young's modulus from 419 MPa to 646 MPa and weakened (irreversible) plastic deformations. The compressive strength of the matrices (178 MPa to 251 MPa), indicating that it is in the range of values for native cortical bone (170–193 MPa). The biocompatibility and lack of toxic effect give the composites a perspective for osteoplastic application, especially with the use of ZnO nanoparticles.

Nanocomposite scaffolds based on polyvinyl alcohol@chitosan@modified clay@HAp were developed.^186^ HAp used in these 3D scaffolds was synthesized from a chicken femur, and Cloisite 30B clay nanoparticles were modified by graphene oxide and Fe_3_O_4_ nanoparticles to strengthen their mechanical properties. The addition of HAp particles and modified clay favored mineralization on the surface of the scaffolds, and the compressive strength was 9.31 MPa, with a porosity of 75% and a pore size of 50 nm, which means that the scaffolds were within the cancellous bone. The final swelling was 1790%, which is the favorable amount for bone structure. The materials did not show toxicity, favoring cell viability.

Chitosan@graphene oxide@HAp@chlorhexidine digluconate membranes were obtained and used as antimicrobial material.^187^ The results were positive for tensile strength tests for the membranes, which ranged from 32.06 to 33.72 MPa. Antimicrobial evaluation showed a significant inhibition of biofilm growth for all membranes. The drug release was 55.6% in 72 h. Therefore, synthesized membranes were considered a promising material for bone regeneration in periodontal lesions.

Hydrophilic scaffolds based on chitosan, xanthan gum, graphene oxide, and HAp associated with mesenchymal stem cells for application in regenerative dentistry.^188^ The use of graphene oxide significantly increased the compressive strength compared to other compositions. The bioactivity test indicated the precipitation of HAp crystals on the scaffold surface. The MTT test showed high cell viability in the scaffolds, indicating that they are promising for application in regenerative dentistry once they presented favorable morphological characteristics and mechanical and biological properties to the regeneration process.

Designed to coat stainless steel implants, a nanocomposite coating was developed for 316L stainless steel implants (SS316L) based on HAp@chitosan@gelatin and functionalized with reduced graphene oxide (rfGO) by electrophoretic deposition at 80, 100 and 120 V to improve the mechanical properties of implants.^189^ The presence of HAp favored the porosity of the coating; however, it reduced the cracks. The corrosion was affected by the morphology of the coating and therefore by the interactions formed between the coating components.

Incorporation of pure superparamagnetic iron oxide nanoparticles into scaffolds results in the creation of magnetic structures for magnetic hyperthermia and bone regeneration. Therefore, HAp@chitosan@poly(vinyl alcohol) scaffolds were obtained.^190^ The results indicated that the scaffolds containing 3.77 and 5.54% by mass of iron oxide nanoparticles achieved temperature increases between 6.6 and 7.5 °C in magnetic hyperthermia tests. *In vitro* studies using human osteosarcoma Saos-2 cells indicated that iron oxide significantly stimulated cell adhesion, proliferation, and alkaline phosphatase expression compared to samples without the magnetic particles.

A coating based on HAp, chitosan, and suspensions containing ZrO_2_ and MgO was obtained on a titanium substrate by electrophoretic deposition in a reverse electric field.^191^ Ca, Zr, and Mg were uniformly distributed in the direction of the thickness of the coating. With the addition of ZrO_2_ and MgO, the contact angle was significantly reduced from 52.5° to 27.3°. The bond strength of the samples obtained, and the titanium substrate was up to 29.3 MPa, exceeding the standard requirements for bond strength of HAp coating implants (15 Mpa, ISO13779-2) in addition to superior electrochemical stability and strong corrosion strength when immersed in simulated body fluid. Furthermore, after immersion in simulated body fluid for 24 days, the surface was completely covered with carbonated HAp, and the Ca/P ratio of the coating increased from 1.67 to 1.99. Furthermore, the antibacterial evaluation against *Escherichia coli* and *Staphylococcus aureus* reached 98.2% and 86.3%, respectively.

HAp@TiO_2_ nanoparticles^192^ are commonly used as a reinforcement agent for biological materials. To improve the application in biological systems, hydrogels based on alginate, chitosan, gelatin, TiO_2_, and HAp were synthetised. According to the authors, the compressive strength and modulus were significantly improved with the increase in TiO_2_ nanoparticles. Furthermore, the addition of TiO_2_ nanoparticles could effectively regulate swelling, *in vitro* biodegradation and the biomineralization, and biological activity of the hydrogels, exhibiting good cell adhesion, activity, and proliferation. Furthermore, TiO_2_ nanoparticles improved the relative alkaline phosphatase activity of cells, effectively promoting cell differentiation.

**Figure 13:**
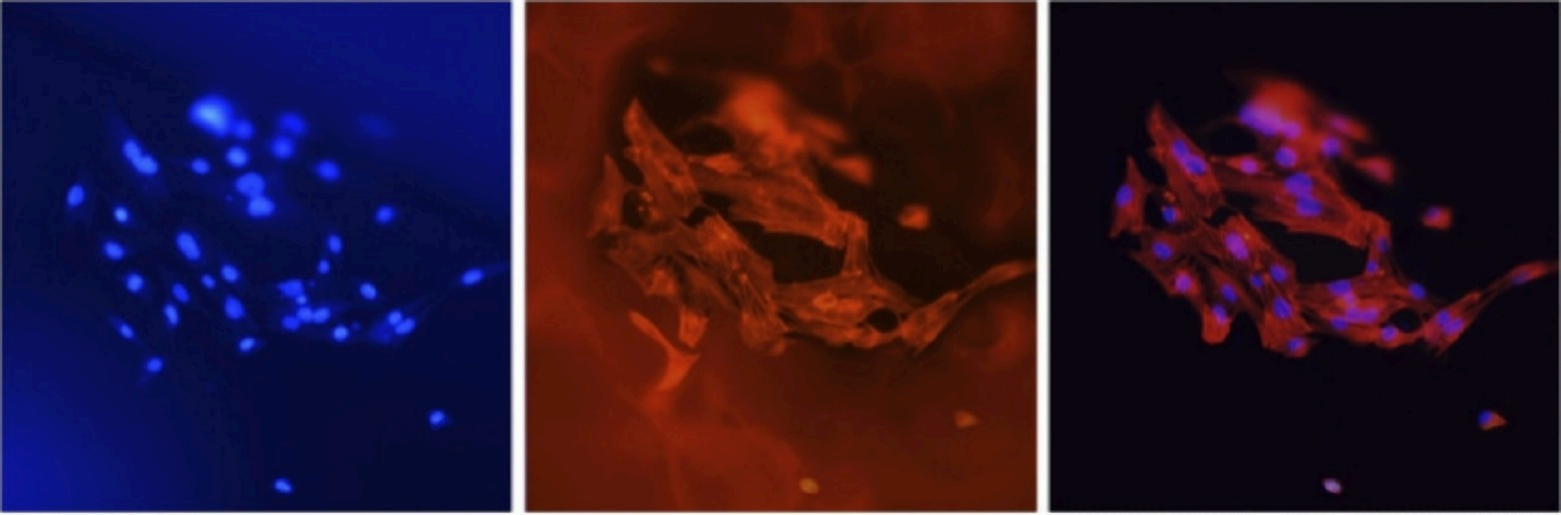
Cells (MC3T3-E1) attached to the scaffolds. Blue fluorescence shows the nuclei stained with DAPI, and red fluorescence shows the cellular F-actin stained with FITC-phalloidin. The magnification was ×400.^193^ Reprinted with permission from ref (193), Copyright 2022, Frontiers in Chemistry.

A new degradable gelatin@carboxymethyl chitosan bone scaffold loaded with nano-HAp and β-TCP, and freeze drying combined with stir foaming resulted in highly connected macropores.^193^ The scaffolds were cultured in vitro with MC3T3-E1 cells (Figure 13), showing that osteoinduction and osteoconduction increased with the phosphate content.^193^

**Figure 14:**
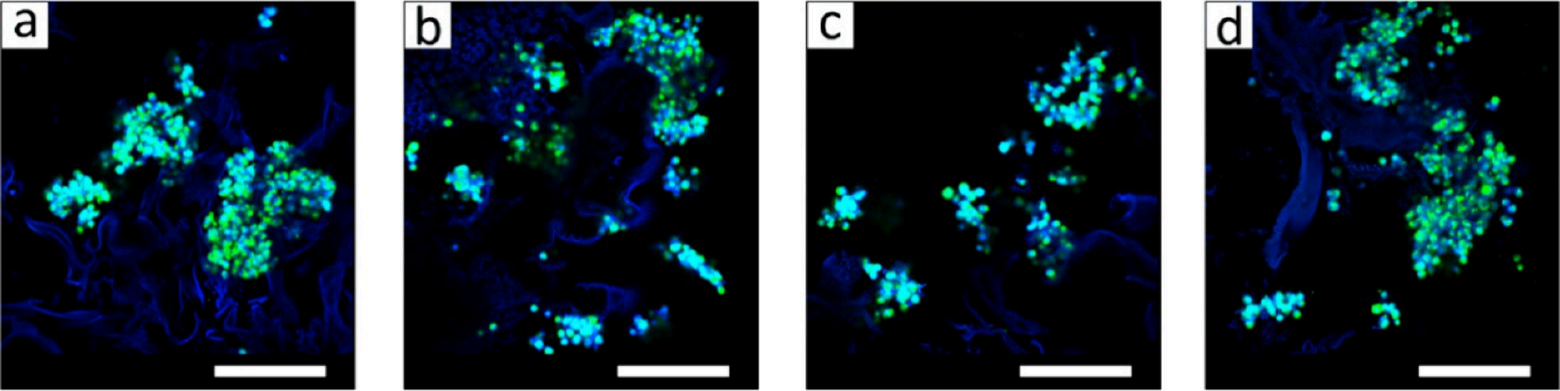
CLSM images of the hyaluronic acid/chitosan hydrogel samples with the L929 cells after cultivation for 1 week. The Hyal/Ch sample was without hydroxyapatite nanoparticles (a); the Hyal/Ch/nHAp-1 (b), Hyal/Ch/nHAp-5 (c), and Hyal/Ch/nHAp-10 (d) samples were loaded with 1, 5, and 10 wt % of hydroxyapatite nanoparticles, respectively. Scale bar is 200 μm.^194^ Reprinted with permission from ref (194). Copyright 2022, MDPI.

Macroporous hydrogels based on a hyaluronic acid@chitosan@polyelectrolyte complex loaded with homogeneously distributed nano-HAp were evaluated *in vitro* using mouse fibroblasts (L929), osteoblast-like cells (HOS), and human mesenchymal stromal cells (hMSC).^194^ Cell morphology and localization within the hydrogels was studied by Confocal Laser Scanning Microscopy. Cell viability was dependent on the nano-HAp content and was evaluated by MTT assay after 7 days of culture on the hydrogels. An increase in nano-HAp loading in a range of 1–10 wt % resulted in an improvement in cell growth and proliferation for all hydrogels (Figure 14). Maximum cell viability was obtained for the sample with 10 wt % nano-HAp, while a minimal cell number was found for 1 wt % nano-HAp).

Antimicrobial activity was evaluated and anti-inflammatory activity with protein denaturation and proteinase inhibition assays, in addition to cell viability of pectin@HAp nanocomposites.^138^ The antimicrobial activity of these nanoparticles against *Pseudomonas aeruginosa*, *Staphylococcus aureus*, and *Klebsiella pneumonia* resulted in inhibition zone values of 4–12 mm, 3–10 mm, and 4–13 mm, respectively. The denaturation of a protein involves the disorganization of the secondary and tertiary structures of proteins created by the application of force, leading to the loss of their biological capacity, being a well-known cause of inflammation. The denaturation mechanism included the change in disulfide and hydrogen bonding, along with the change in hydrophobic and electrostatic attraction forces.^138^

Neutrophils, which release mediators such as serine proteinases located in lysosomes, play an important role in the pathophysiology of inflammation and thus play an important role in the progression of tissue damage. Therefore, the medications used to inhibit these proteinases have anti-inflammatory properties. The proteinase activity observed for the obtained nanocomposites was over 60%, and it is believed that proteinase inhibitors reported as anti-inflammatory drugs work by inhibiting neutral proteinases generated by polymorphonuclear leukocytes, rather than directly inhibiting them directly.^138^ The effect of the nanocomposites obtained on the viability of the MG-63 cell line was also evaluated. The results revealed a dose- and time-dependent decrease in cell viability in both cell lines compared to the control group. The results indicate that both high-concentration samples have favorable properties for adhesion and dissemination of MG-63 cells.^138^

An *in vitro* cell study using MG-63 cells, which are similar to human osteoblasts, demonstrated that the inorganic size and crystallinity of pectin@Sr-doped HAp played a vital role in the regulation of osteogenesis. The study suggested that the synchronization of low pectin concentration (0.5 wt%) and high Sr^2+^ substitution in HAp (30 mol%) offered the desired microhardness and osteogenic properties in vitro to emulate natural bone.^24^

Cathodic electrophoretic deposition was used to fabricate novel chitosan@pectin@HAp porous nanocomposite coatings on Ti6Al4V substrates. The Rietveld refinement indicated variations in structural changes resulting from reducing the applied voltage from 30 to 10 volts. Deposits obtained at 10 volts after 1 h of presedimentation exhibited a dense microstructure with a narrow pore size distribution compared to others. The wettability and adhesion strength of coated Ti6Al4V improved with increasing HAp content. The release from the composite coating confirmed a 40% initial release, 50% semi stable release, and 10% residual release. A thick and uniform layer of porous apatite was formed in Ti6Al4V after 21 days of immersion in simulated body fluid.^156^

##### 2.3.2.3 Other Biomedical Applications

For example, collagen@alginate@HAp in scaffolds used in bone tissue engineering was evaluated for the release of Ca^2+^ ions, in which the behavior of the hydrogel was pH dependent.^195^ The results showed that the pH value decreased drastically in the first 5 min after adding the polysaccharide, while the presence of phosphate had the opposite effect, due to the consumption of released hydrogen ions present in the polysaccharide. Therefore, the amounts of polysaccharide and phosphate were fixed as a way to control the pH value between 6.8 and 7.2 during gelation, which would be suitable for drug administration and other biomedical applications. The results showed that the systems remained in the physiological situation. The reason for the increase in pH value due to the addition of HAp to hydrogels may be the partial consumption of hydrogen ions present in the polysaccharide.^195^

Sodium alginate@chitosan@HAp hydrogel containing different amounts of HAp was synthesized using gamma radiation as cross-linker to be utilized for oral delivery drug.^196^ The efficiency of hydrogel samples as a drug delivery of doxorubicin. The *in vitro* drug release behavior of doxorubicin from the nanocomposite was studied at pH 7.4 and pH 5 in 24 h. The drug release was pH dependent, and samples showed a higher release at pH 5.^196^

Recently, the fabrication of a scaffold from biomaterials has been increased due to the lack of adequate natural bone for grafting. A chitosan@alginate@polyamide@HAp synthetic scaffold was fabricated using the thermally induced phase separation technique. The scaffold was cross-linked with either a chemical cross-linker (calcium chloride, 2-hydroxyethyl methacrylate, or glutaraldehyde) or a physical cross linker (gamma irradiation). The cross-linked scaffolds were characterized based on physicochemical properties, cytotoxicity, and biocompatibility. Porosity and density of scaffolds were 83.33–92.14%, and 0.241–0.335 g/cm^3^, respectively. The swelling ratio for the same scaffolds was 108–149% after 72 h of observation. Brine shrimp cytotoxicity and an RBC biocompatibility assay confirmed the nontoxic nature of scaffolds. The gamma irradiation chitosan@alginate@polyamide@HAp scaffold was tested for bone regeneration in the rabbit mandible defect model. Histological analysis revealed the regeneration of new bone and restoration of bone defect at the site of injury.^197^

Scaffolds based on alginate, chitosan, and F-doped HAp were also evaluated for their release and antimicrobial efficacy.^148^ According to the authors, antimicrobial studies indicated that materials obtained from the addition of biopolymers have better antifungal and antibacterial activities compared to materials without modification; also, greater activity was observed against *Staphylococcus aureus* with an inhibition zone of 47 mm, while *Escherichia coli* had an inhibition zone of 38 mm.

Composites based on Er-doped HAp for the development of luminescent chitosan@HAp films were proposed for action as an antimicrobial material and as an implant material or fluorescent cell regeneration material. Chitosan films were flexible and biochemical tests indicated excellent antimicrobial, bioactive, and fluorescent properties under in vitro conditions.^87^

The extraction of palm pectin for the production of HAp composites.^77^ The results indicated that the concentration of pectin has an influence on the purity, crystallinity, particle size, and morphology of the HAp nanoparticles. As the concentration of pectin increased in relation to HAp, a tendency toward amorphism was observed for higher pectin concentration. The *in vitro* bioactivity of the HAp nanoparticles synthesized with pectin indicated the formation of a layer of porous apatite on the surface of the spheres after 14 days of immersion in the simulated fluid. HAp nanoparticles showed better antimicrobial activity for the pathogenic test of *S. aureus* and bacterial strains of *E. coli* and *C. albicans* compared to nanoparticles without pectin.^77^

Cell adhesion and proliferation of the compound obtained from HAp and pectin extracted from jackfruit peels were evaluated in vitro.^198^ Morphological analysis showed that osteoblast adhesion to the bionanocomposite increased from day 5, and, from day 7, cells adhered to the entire surface. Cell proliferation increased considerably with culture time, as cells developed lamellipodia and filopodia. The compound considerably increased the biological reaction to osteoblasts.^198^

HAp nanoparticles were synthesized using different concentrations of pectin (0.1, 0.5, and 1%) from *P. biglobosa* pulp as a green template.^199^ The authors confirmed the formation of the biomaterial, and, according to the structure and morphology of the synthesized hydroxyapatite, it was possible to observe that at a low concentration of pectin there is the formation of small and less agglomerated particles with nanoparticles that present a crystallite size ranging from 17.5 to 26.3 nm, favoring inhibition of the growth of *E. coli* bacteria. In fact, low-crystal-level HAp has a high capacity to be resorbable *in vivo*. At low concentrations of pectin, HAp particles with low crystallinity and high purity were produced because the pectin molecules influence the size of the HAp crystals, as the polysaccharide allows the formation of a three-dimensional network. The study suggested that the pectin from *P. biglobosa* pulp can serve as a green model for the production of HAp nanoparticles, which are considered promising and versatile bioactive materials that target applications in enzyme technology, biomedical engineering, and tissue engineering.^199^

*In vitro* studies of biomineralization, cell viability, antimicrobial activity, protein adsorption, and biodegradability studies of composites based on pectin@HAp were performed.^128^ However, other forms of use of this composite have been reported, such as the case of a hydrogel produced from commercial HAp and pectin including the rheological properties, determination of the injectability of this gel, in vitro dissolution, and cytotoxicity for applications in tissue engineering.^128^

According to the authors, the gelation kinetics of the hydrogels occurred between 9 and 50 min after preparation. Among the tunable rheological properties, varying the composition of the hydrogels, the storage modulus, provides quantitative information on the hydrogel ability to resist deformation that may occur during injection or after implantation into neighboring tissues. Based on the results of the indirect cytocompatibility assay, the hydrogel formulation favored immobilization of a model cell line (L929 fibroblasts) that allowed the gel network to be sufficiently permeable to oxygen and nutrients, supporting cell viability. A slight decrease in cell viability was observed in the first hour after immobilization, and then it was maintained almost constant until the end of the experiment (24 h), indicating that the pectin-hydroxyapatite hydrogels provide a suitable environment for cells. In general, these systems are suitable supports for cell immobilization for tissue regeneration applications.^128^

One way to improve the characteristics of synthetic HAp is ionic substitutions, including cationic exchange as reported,^7^ where Ce^3+^ doped HAp and biopolymer gellan gum were used in scaffold production. The scaffolds had mechanical resistance to compression of 25.38 MPa and 28.87 MPa, respectively. When comparing the values presented by the scaffolds and the values of mechanical resistance to compression of the bones of the human body, both scaffolds presented sufficient mechanical resistance to be applied in the replacement of cancellous bone, since the mechanical resistance of cancellous bone varies between 4 and 12 MPa. Regarding compression resistance, the mechanical equivalence of the scaffold with respect to cancellous bone is very important, as the cancellous allograft is recommended by surgeons as the third best option for clinical bone replacement and is considered by experts as the gold standard.^7^

The sodium alginate@montmorillonite@HAp^141^ composites have biological potential, such as antibacterial and antioxidant. The antibacterial potential of the composite was evaluated according to the zone inhibition method. The antibacterial results showed better elimination in the bacterial zone for the polysaccharide-containing composite. The presence of the composite significantly affected the bacterial cultures *S. aureus* and *P. aeruginosa*, with maximum inhibition being up to 34 mm against *S. aureus*.^141^ Free radicals that naturally form are highly unstable molecules in human cells. Consequently, antioxidant substances have been used to prevent cellular damage in the human cell. Enzymatic and non-enzymatic reactions affect human cells for the formation of free radicals, which causes them to occur continuously. The results obtained from the inhibition of free radicals in the prepared composites were compared with the standard medicine, vitamin C. The antioxidant action for the maximum concentration of the material obtained was 78.48 ± 2.5%, and 83.89 ± 2.7% for vitamin C.^141^

The antibacterial activity of the pectin@nano-HAp composite was evaluated,^139^ determining the inhibition zone values during the diffusion method of the agar well. Inhibition zone values were evaluated for the different concentrations of the composite obtained (0.05–0.20% by weight). The values were 16–20 mm against *S. aureus* and 15–22 mm against *E. coli*. Erythromycin at 0.3 mg mL^–1^ was used as a positive control and had an inhibition zone of 25 mm.^139^ According to the authors, the antibacterial activity of the composites was attributed to the formation of the calcium carboxylate complex, where an interaction occurs between calcium derived from eggshells and carboxylic groups present in the structure of pectin. Therefore, the calcium carboxylate group may be responsible for the antibacterial nature of the composite.^139^

The biofilm formation component of the composite was evaluated and showed biofilm inhibition values of approximately 67% and 74% for *S. aureus* and *E. coli*, respectively. Therefore, the material can prevent the development of biofilms by pathogens. The composite showed the ability to reduce the formation of exopolysaccharides produced by *S. aureus* and *E. coli*. Consequently, the composite obtained has the potential to prevent biofilm-related problems in bone transplants or dental implants safely, without employing chemicals that raise consumer concerns.^139^

Carboxymethyl chitosan@sodium alginate@HAp hydrogel showed antibacterial efficiencies of 82% against *S. mutans* and 93% against *P. gingivalis*. The hemolysis rate was less than 5%, and good biocompatibility was observed. The hydrogel was efficient in the preparation of tissue engineering structures.^200^

A biomatrix composed of collagen, carrageenan, and nano-Hap modified with lanthanum oxide was explored as a proangiogenic and osteogenic biomaterial for bone tissue repair.^201^ The biomatrix presents better physical and biological stability, as observed in studies of proteolytic degradation and thermal stability. The addition of lanthanum oxide nanoparticles improved osseointegration along with simultaneous activation of proangiogenic properties to act as a bone mimic material. The synthesized biomatrix achieved capillary migration into the bone microenvironment due to a minimal level of reactive oxygen species and superior cytocompatibility. The biomatrix positively regulated the expression of the VEGF, VEGF-R2 genes in endothelial cells and osteopontin, osteocalcin in osteoblast cells, respectively. *In vivo* analysis of hard tissue repair performed on a rat model showed complete healing of bone defect healing in eight weeks with biomatrix application of the produced compared to biomaterials based on individual components.^201^

As a way to increase the applicability of alginate hydrogel in tissue engineering, oxidized sodium alginate@polyacrylamide@gelatin hydrogels were developed through the interpenetrating network approach with d-glucono-delta-lactone@HAp as an endogenous ionic cross-linking agent for oxidized alginate and *N*,*N*′ -methylenebisacrylamide as a chemical cross-linking agent for polyacrylamide, followed by coating with gelatin. Then, TiO_2_ nanoparticles were added as a reinforcing agent to the alginate@polyacrylamide@gelatine hydrogel matrix to construct alginate@TiO_2_@polyacrylamide@gelatine hybrid composite hydrogels. The addition of TiO_2_ nanoparticles to the alginate@TiO_2_@polyacrylamide@gelatine composite hydrogel could effectively regulate the porosity, mechanical properties, swelling rate, biodegradability *in vitro*, and biomineralization of the composite. Furthermore, composite hydrogels had the best biocompatibility, indicating that the addition of TiO_2_ nanoparticles can effectively promote cell adhesion, proliferation, and differentiation.^202^

Due to stress-protective effects, traditional Ti alloy scaffolds have a high modulus of elasticity, which can promote bone laxity and disintegration around the implant, increasing the possibility of a second surgery. On the contrary, 3D printed porous Ti alloy scaffolds can reduce scaffold weight while improving biocompatibility.^203^ Furthermore, the porous nature of Ti scaffolds allows for bone tissue and strong pore connectivity, which can improve nutrient absorption. However, pure titanium alloy implants may fail due to inadequate osseointegration; therefore, adding a coating to the implant surface is an effective technique to improve implant stability. In this sense, the HAp@chitosan@tannic acid@Cu^2+^ composite was prepared on the surface of scaffolds containing a 3D printed porous Ti alloy by electrophoretic deposition. Composite coating has better antibacterial properties and cytocompatibility as well as lower cytotoxicity, and the alkaline phosphatase assay indicated that the coating resulted in good potential for osteogenesis.^203^

**Figure 15:**
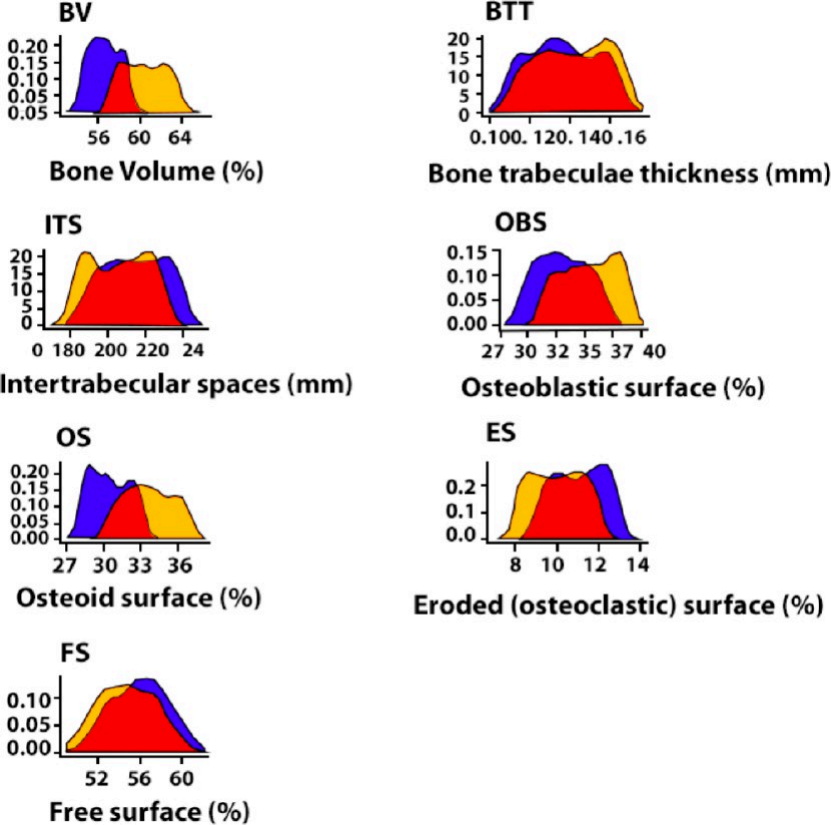
Distribution density of histomorphometric parameters in the area of postoperative bone tissue defect in rats with sub-compensated diabetes mellitus in control group 3 (collagen) and experimental group (CH-SA-GA) 4 weeks after modeling the disease. The ordinate axis shows the density of bone formations. Collagen—blue fragment of the graph, CH-SA-HA-yellow fragment of the graph.^204^ Reprinted with permission from ref (204), Copyright 2023, MDPI.

A polyelectrolyte construct composed of high molecular ascorbate of chitosan, chondroitin sulfate, sodium hyaluronate, heparin, serum growth factor, sodium alginate, and nano-HAp was used to increase the efficiency of early bone formation in a critical size in *diabetes mellitus*.^204^ Studies were conducted on five groups of white female Wistar rats: group 1 - regeneration of a bone defect in healthy animals under a blood clot; group 2 - regeneration of a bone defect under a blood clot in animals with diabetes mellitus; group 3 - bone regeneration in animals with diabetes mellitus after filling the bone cavity with a collagen sponge; group 4 - filling of a bone defect with a chitosan@sodium alginate@HAp complex in healthy animals; group 5 - filling of a bone defect with a chitosan@sodium alginate@HAp construct in animals with diabetes mellitus. Chitosan@sodium alginate@HAp cosntruct in diabetes mellitus created a high efficiency of bone regeneration and significantly compensates for the level of osteogenesis (Figure 15).

Given the vast number of studies found in the literature that produced and used HAp nanocomposite polysaccharide systems with different applications, some of these studies are briefly summarized in Table 4.

**Table 4:** Summary HAp@polysaccharides (Nano)composites and Their Applications Reported in the Literature

Systems	Applications	References
Chitosan@HAp composite obtained by electrodeposition	*In vitro* study aiming at biomedical applications	(205)
Steel coating with chitin, chitosan, and HAp	Corrosion resistance assessment	(172)
Composite produced from HAp and chitosan obtained from natural sources	Assessment of bioactivity	(9)
Porous *scaffolds* produced from alginate, HAp, and lactose modified chitosan	Evaluation of healing properties, osteoinductive, and osteoconductive properties from stem cells	(206)
Chitosan@alginate hydrogel and simultaneous incorporation of HAp containing (1–34) PTH, calcium and phosphorus regulatory hormone	Bone regeneration	(207)
Alginate@chitosan@HAp scaffolds	*In vitro* and *in vivo* assays for cartilage regeneration, cell viability and fixation, and antimicrobial evaluation	(208)
Alginate@chitosan@gelatin hydrogel and TiO_2_@HAp nanoparticles		(192)
Alginate@ZnO@HAp cross-linked by chitosan and Ca^2+^, Zn^2+^, and Cu^2+^	Creation of dressings	(209)
Alginate@HAp	Adsorption of Lactobacillus	(210)
Carboxymethylcellulose@HAp s*caffolds*	Orthopedic application	(136)
Electrophoretic deposition chitosan@bioglass@HAp as mesoporous coatings on titanium implant	Vancomycin loading and release	(211)
Gelatin@chitosan@carbonated HAp films modified with tetraethyl orthosilicate as crosslinker	Bone-grafting particles to loading and release local of metronidazole antibiotics in treatment the infection and stimulate the bone growth and regeneration	(212)
Sr^2+^ or Cu^2+^ α-TCP as precursor for Ca^2+^ deficient HAp to form cement with photocrosslinked methacrylated alginate thin film	Antibacterial activity, bone formation, and vascular regeneration	(213)
Ti doped HAp@gelatin-pig-skin and Ti doped HAp@alginate- algae	Biomimetic materials, sustainable sunscreen UV-filters	(214)
Chitosan@calcium phosphate matrix containing multiwalled carbon nanotubes, graphene oxide, Fe_3_O_4_	Release of pregabalin	(215)

## 3 Conclusion and Remarks

Polyssacharides@hydroxyapatites (nano)composites are promising materials for biomedical applications, especially as carriers and adsorbents for drugs and bioactive species and body implants. The (nano)composites can be obtained as powder, hydrogel, scaffolds, and membranes. The presence of HAp in (nano)composites reduces the dissolution of polysaccharides, and, consequently, the resulting biomaterials presented better biological properties, such as bioactivity and biodegradability. The use of polysaccharides improved the drug release systems or bioactive molecules of the most diverse types, such as antibiotics, anti-inflammatory drugs, tumor molecules, and molecules with therapeutic properties in places that require slow and prolonged release. Systems such as scaffolds promoted improvements in mechanical and structural properties aimed at applications in tissue engineering such as porosity, compressive strength, rheological properties, and swelling rate. Biological properties include cell viability, cytocompatibility, expansion capacity, water absorption, osteoinduction, and antimicrobial evaluation, among others. Some presented studies carried out evaluations of biological properties; however, *in vitro* and *in vivo* tests need to be expanded.

Faced with a growing production of studies focused on the area of biomaterials and biomedical applications, as observed in this review, some points were identified that still deserve attention. In this sense, some perspectives are presented:

I. It is necessary to combine different polysaccharides such as pectin and HAp or even other calcium phosphates obtained from other natural sources to prepare these systems.
II. Systems based on other inorganic phases, such as clays mineral or even the presence of oxides that are used in the biomedical field, such as ZnO.
III. Another important point is a better understanding of the behavior of drugs in complex systems, such as hybrids containing HAp and other inorganic materials.
IV. A relatively small number of drug release focused on *in vivo* analysis.
V. Characterization on the molecular scale for better understanding of the interactions in the nanocomposites and their influence on the biological properties.
